# Pharmacological treatments for vascular dementia: a systematic review and Bayesian network meta-analysis

**DOI:** 10.3389/fphar.2024.1451032

**Published:** 2024-08-22

**Authors:** Chun Dang, Qinxuan Wang, Yijia Zhuang, Qian Li, Li Feng, Ying Xiong, Yaoheng Lu

**Affiliations:** ^1^ Department of Periodical Press, West China Hospital, Sichuan University, Chengdu, China; ^2^ West China Hospital, West China School of Medicine, Sichuan University, Chengdu, China; ^3^ Department of Neurology, The Second Affiliated Hospital of Harbin Medical University, Harbin, China; ^4^ Department of General Surgery and Regenerative Medicine Research Center, West China Hospital, Sichuan University, Chengdu, China; ^5^ Department of General Surgery, Chengdu Integrated Traditional Chinese Medicine and Western Medicine Hospital, Chengdu, China

**Keywords:** Bayesian network meta-analysis, vascular dementia, treatment decision-making, meta, VAD

## Abstract

**Background:**

Vascular dementia (VaD) is one of the most prevalent, burdensome, and costly forms of dementia. Pharmacological treatment is often the first-line choice for clinicians; however, there is a paucity of comparative information regarding the multiple available drug options.

**Methods and Analysis:**

A systematic review and network meta-analysis were conducted on randomized trials involving adult patients with VaD, sourced from PubMed, the Cochrane Library, EMBASE, Web of Science, OPENGREY, ClinicalTrials.gov, Wanfang Data, and CNKI. The primary outcomes included changes in Mini-Mental State Examination (MMSE) scores, activities of daily living (ADL) scores, and the incidence of adverse reactions. Efficacy and safety of intervention strategies were comprehensively analyzed using forest plots, cumulative ranking probability curves (SUCRA), and funnel plots, all generated with R software.

**Results:**

A total of 194 RCTs comparing 21 different anti-VaD drugs with placebos or no treatment were analysed. Regarding MMSE scores, the five most effective drugs were Butylphthalide, Huperzine A, Edaravone, Rivastigmine, and Memantine. For ADL scores, the top five drugs in efficacy were Huperzine A, Butylphthalide, Tianzhi granule, Nicergoline, and Idebenone. In terms of the incidence of adverse drug reactions, Co-dergocrine Mesylate, Tongxinluo capsule, Butylphthalide, Piracetam, and Oxiracetam demonstrated favourable safety profiles.

**Conclusion:**

This study enhances the understanding of the relative benefits and risks associated with various VaD treatments, providing a valuable reference for clinical decision-making.

**Systematic Review Registration:**

https://www.crd.york.ac.uk/PROSPERO/, identifier registration number.

## Introduction

Vascular dementia (VaD) is widely recognized as the second most prevalent subtype of dementia, after Alzheimer’s disease (AD). VaD refers to dementia resulting from vascular brain injury that encompasses damage to the brain parenchyma, most commonly due to ischemia, infarction, and hemorrhage (Bir et al., 2019). The most recent diagnostic criteria for VaD classify major VaD into four phenotypic categories: subcortical ischemic vascular dementia, poststroke dementia, multi-infarct dementia, and mixed dementia (Chang et al., 2022). Currently, over 50 million individuals globally are affected by dementia, and projections suggest that the number of cases could quintuple by 2055 (Wolters and Ikram, 2019). VaD is responsible for approximately 15%–20% of dementia cases in North America and Europe (Lobo et al., 2000; Rizzi et al., 2014), with estimates reaching about 30% in Asia (Jhoo et al., 2008; Wolters and Ikram, 2018). The aging demographic trend and extended life expectancy have significantly exacerbated the incidence of VaD.

The pathogenesis of VaD is linked to chronic cerebrovascular damage stemming from conditions such as cerebral artery disease, atherosclerosis, arteriolosclerosis, microvascular disease, and cerebral amyloid angiopathy (Pathan et al., 2024). Clinically, VaD patients may exhibit behavioral changes, reduced attention and memory capacities, and cognitive deficits (Linh et al., 2022). Without timely and effective intervention, the progression of VaD can severely impair neurological functions, leading to motor, sensory, and perceptual deficits that significantly deteriorate the quality of life for individuals.

“Despite its high prevalence and substantial societal impact, VaD lacks Food and Drug Administration-approved treatments, unlike AD”. Therapeutic options and their efficacy for VaD remain considerably constrained (Tisher and Salardini, 2019). Cholinesterase inhibitors enhance cognition by augmenting the levels of intrasynaptic acetylcholine, a neurotransmitter pivotal for memory and learning (Román and Kalaria, 2006; Chen et al., 2016). Although cholinesterase inhibitors such as donepezil, galantamine, and rivastigmine are sanctioned for AD in the United States and most European countries, their approval does not extend to VaD. However, donepezil has received approval for VaD from regulatory agencies in New Zealand, India, Romania, South Korea, and Thailand. Additionally, Memantine, a non-competitive N-methyl-D-aspartate (NMDA) receptor antagonist, mitigates glutamatergic signalling through NMDA receptors. Memantine is approved in Argentina, Brazil, and Mexico for the management of VaD (Burns et al., 2006; Johnson and Kotermanski, 2006). Furthermore, numerous therapeutic agents demonstrate potential in the treatment of VaD, encompassing antioxidants, anti-inflammatory therapies, neuronutritional supplements, neuroprotective agents, and nootropic drugs (Chang et al., 2022).

Additionally, “Chinese medicine treatment of vascular disease guidelines for the clinical application of dementia (2020 edition)” issued in China advocate the use of proprietary Chinese medicines for VaD treatment, highlighting agents such as butylphthalide, tongxinluo capsule, Edaravone, and tianzhi granule. In clinical practice, various herbal medications are widely used as alternative treatments to conventional pharmacotherapies (Chan et al., 2018; Kim and Kang, 2020), providing a complementary approach in the therapeutic landscape of VaD (S. P. T. f. t. G. o. C. A. o. P. C. M. i. t. T. o. V., 2021). In practice, a plethora of Chinese patent medicines are used as alternative treatments to conventional pharmacotherapies (Chan et al., 2018; Kim and Kang, 2020). However, discerning the comparative efficacy and safety of these diverse pharmacological therapies presents a considerable challenge. For clinicians tasked with selecting from different treatment options, synthesizing the available evidence on VaD from extant trials is crucial to ascertain the relative effects and safety of the treatment therapies.

Network meta-analysis (NMA) is an advanced statistical technique used to assess and compare the efficacy and safety of multiple therapeutic interventions concurrently. By integrating both direct comparisons between treatments and indirect comparisons across trials sharing common comparators, NMA facilitates a robust synthesis of evidence. This methodology extends the scope of traditional pairwise meta-analysis, allowing for a comprehensive ranking of competing treatments based on their effectiveness and safety, thereby supporting evidence-based clinical decision-making.

This study is designed to undertake a series of comparisons between various pharmacological therapies for VaD treatment. It aims to extensively evaluate existing evidence and provide a robust foundation for clinical decision-making, enhancing the understanding of the comparative benefits and risks associated with each pharmacological intervention.

## Methods

The systematic review was meticulously conducted adhering to the PRISMA Extension Statement for Reporting Systematic Reviews with Network Meta-Analyses (Hutton et al., 2015), with the study protocol registered in the PROSPERO database (CRD42024521910).

### Search strategy and data retrieval

Comprehensive searches were executed across multiple electronic databases from their inception through March 2024, including PubMed, the Cochrane Library, EMBASE, Web of Science, OPENGREY, ClinicalTrials.gov, Wanfang Data, and Chinese National Knowledge Infrastructure (CNKI). Searches of the grey literature were also undertaken. The objective was to identify peer-reviewed studies that assess the efficacy and safety of pharmacological interventions for VaD. The detailed search methodologies are delineated in Supplementary Appendix 1.

We scrutinised the reference lists of all pertinent articles, study reports, and conference proceedings, and conducted searches of unpublished literature to capture additional relevant studies, thereby minimising the risk of omissions. Following the removal of duplicate entries, two investigators (Dang et al., 2023) independently screened titles and abstracts to identify studies potentially meeting the inclusion criteria. These articles were further assessed for eligibility by the same researchers. Any disagreements were resolved through discussion or, if necessary, consultation with a third researcher (Qian Li). Consultations with subject matter experts were sought as required.

#### Study selection

The research question was meticulously structured using the PICOS (Population, Intervention, Comparison, Outcome, Study design) framework. Population: Inclusion was limited to patients diagnosed with VaD; Intervention: patients undergoing treatment with a single pharmacologic agent; Comparison: participants were compared across groups receiving a single drug, placebo, or no treatment for VaD; Outcome: primary outcomes assessed included the Mini-Mental State Examination (MMSE) score, activities of daily living (ADL) score, and the incidence of adverse reactions. Study Design: randomized controlled trials (RCTs).

The inclusion criteria were as follows: (Bir et al., 2019): Patients conforming to established diagnostic criteria for VaD, as outlined by the Diagnostic and Statistical Manual of Mental Disorders, the National Institute of Neurological Disorders and Stroke (Wen et al., 2022). (Chang et al., 2022) Patients receiving monotherapy for VaD (Wolters and Ikram, 2019). Studies that reported on efficacy and safety outcomes, specifically MMSE score, ADL score, and incidence of drug-related adverse reactions.

The exclusion criteria were as follows: (Bir et al., 2019): Studies involving patients diagnosed with AD or other subtypes of dementia caused by different etiologies (Chang et al., 2022). Patients undergoing multiple therapeutic interventions (Wolters and Ikram, 2019). Individuals presenting with severe neurological deficits or significant medical conditions such as visual impairment, aphasia, hearing loss, and malignancies (Lobo et al., 2000). Studies lacking primary outcome data.

### Data extraction

Data extraction from the included RCTs was conducted independently by two reviewers, Dang et al., 2023. The data extracted encompassed detailed study characteristics, including the first author, year of publication, participant demographics (sex, age, sample sizes, follow-up duration), details of the intervention group (intervention measures, dosage, administration route), and control group characteristics (control measures, dosage, administration route). Additionally, outcome measures were recorded. Discrepancies encountered during the data extraction process were adjudicated by a third reviewer, Qian Li, through a consensus discussion involving all reviewers. In instances where data were missing or incomplete in the published articles, efforts were made to contact the corresponding authors directly to request the original data. This approach ensured a comprehensive and accurate data compilation critical for subsequent analyses.

### Outcome measures

The efficacy of treatments was evaluated through cognitive function assessments using the MMSE scores, and functional capabilities via the ADL scores. The incidence of drug-related adverse reactions was established as the primary safety outcome indicator. The MMSE is the most extensively utilized neuropsychological scale for screening clinical cognitive functions, effectively reflecting the cognitive status and characteristics of patients (Ciesielska et al., 2016). Given that cognitive impairment is a hallmark symptom of VaD, it was selected as the primary efficacy outcome for this review. Additionally, the deterioration in the ability to perform ADL, a prominent symptom of VaD, was chosen as a secondary efficacy outcome measure (Mlinac and Feng, 2016). The incidence of adverse reactions, defined as the proportion of patients experiencing at least one adverse event relative to the total number of patients in both the intervention and control groups, serves as a globally accepted metric for assessing treatment safety (Patton and Borshoff, 2018).

### Bias assessment

Two independent reviewers, Dang et al., 2023, rigorously evaluated the identified trials. The Cochrane Collaboration’s Risk of Bias Tool (RoB 2.0) was employed to assess the risk of bias in the included RCTs. Any discrepancies between the reviewers were referred to a third reviewer, Qian Li, and were resolved through comprehensive discussion among all reviewers. This process ensured a robust and unbiased evaluation of the studies, maintaining the integrity and reliability of the review.

### Data analysis

A NMA was conducted for each collected outcome within a Bayesian framework. This analysis was predicated on the assumption that the between-study variance (τ^2^) was homogenous across all pharmacological interventions. The principle of transitivity was assessed by examining potential efficacy modifiers, alongside a thorough review of all outcomes and characteristics of participants (Mills et al., 2013). To ensure the consistency across the entire network, a design-by-treatment interaction model was utilized (Chaimani et al., 2013; Jackson et al., 2016). The efficacy and safety of different pharmacological treatments for VaD were quantified using odds ratios (OR) or the logarithm of OR, along with their respective 95% confidence intervals (CI). Due to the presence of between-study heterogeneity, a random-effects model was deemed the most suitable and prudent approach (Dias et al., 2013). Statistical heterogeneity was evaluated using the I^2^ statistic, and the τ^2^ test was employed to ascertain the extent of heterogeneity for each outcome. Publication bias and the impact of small sample sizes were assessed using funnel plots (Higgins and Thompson, 2002). In the Bayesian hierarchical model frameworks, the Markov chain Monte Carlo (MCMC) estimation method was utilized, employing four chains to compute the median treatment effects and 95% CIs. The number of tuning iterations was set at 50,000, with the simulation iterations totaling 100,000 (Gelman and Rubin, 1996). Network diagrams were generated to depict the network geometry and node connectivity visually. Each intervention’s efficacy was ranked using the Surface Under the Cumulative Ranking curve (SUCRA), where higher SUCRA values indicate a greater likelihood of superior therapeutic outcomes. Model convergence was evaluated by visually inspecting the iteration plots and applying the Gelman-Rubin method to assess the potential scale reduction factor. The model fit was appraised by calculating the deviance information criterion (DIC), which combines the posterior mean of the residual deviance with the leverage pD (Salanti et al., 2011). All statistical analyses, including direct and indirect comparisons within the network, were conducted using R software, version 4.3.1 (R Foundation for Statistical Computing, Shanghai, Asia), employing the “gemtc 0.8–2″ and “JAGS” (version 3.5.3) packages (Neupane et al., 2014; Dang et al., 2024).

## Results

### Study identification

**FIGURE 1:**
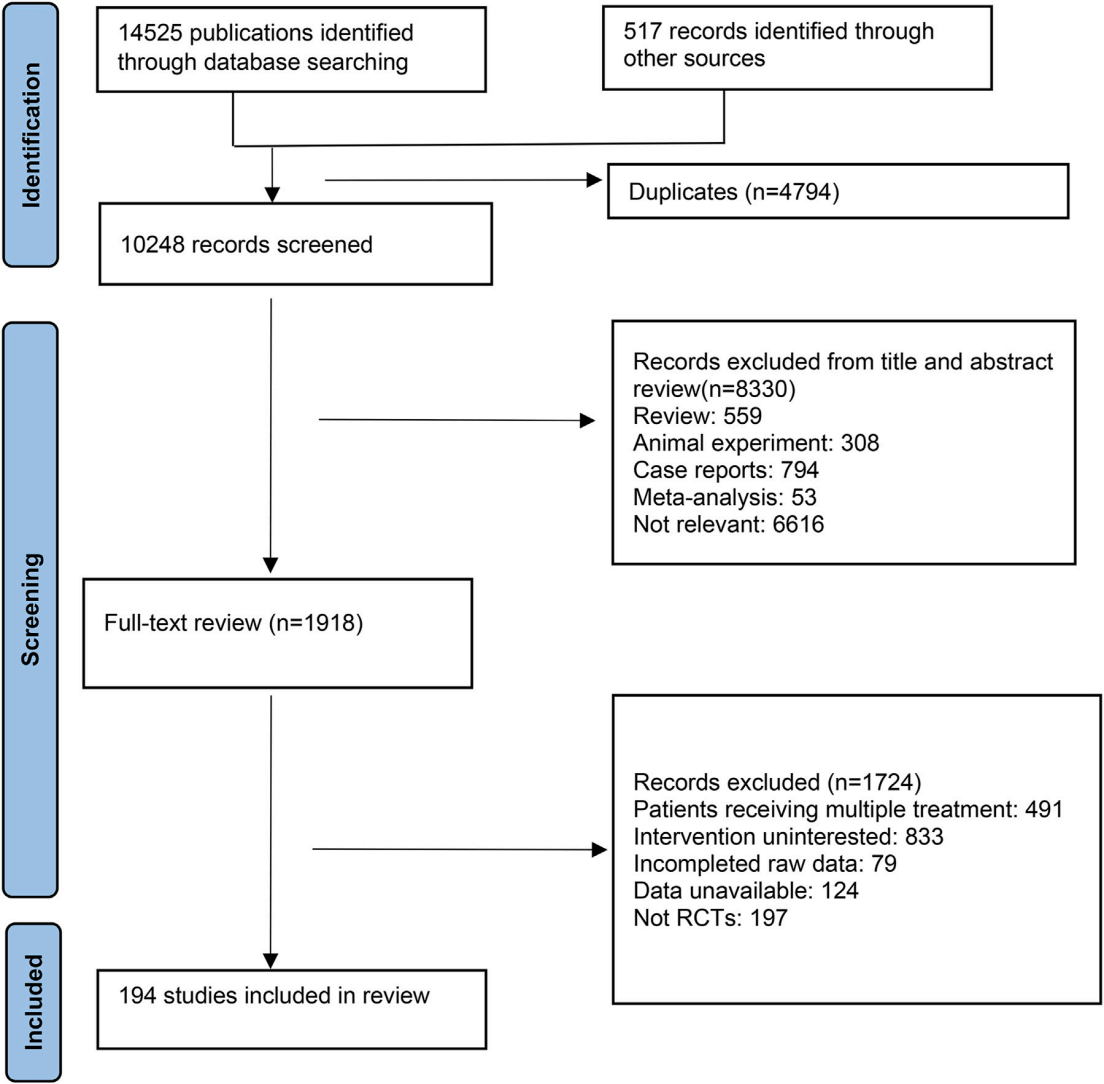
PRISMA flow diagram.

In this study, database searches identified a total of 14,525 publications, supplemented by an additional 517 records from other sources. After eliminating 4,794 duplicate references due to overlapping database coverage, 1,918 studies were selected for further review based on their titles and abstracts. Of these, 1724 studies were excluded for the following reasons: 491 reported multiple treatments, 833 did not investigate the interventions of interest, 79 lacked complete raw data, 124 had unavailable data, and 197 were not RCTs. Ultimately, 194 RCTs met the inclusion criteria and were incorporated into this NMA. The detailed selection process is depicted in Figure 1.

### Characteristics of the included studies

In total, 194 RCTs comparing 21 different anti-VaD drugs with placebos or no treatment were analyzed. The sample sizes in the intervention groups ranged from 8 to 648 participants. Specifically, 8 RCTs on Idebenone included 318 patients and 295 controls. Twenty-seven RCTs on Oxiracetam involved 1,223 patients and 1,204 controls. Thirty-seven RCTs on Donepezil encompassed 4,617 patients and 4,398 controls. Three RCTs on Galantamine included 852 patients and 646 controls. Eight RCTs on Nicergoline consisted of 325 patients and 328 controls. Twenty-two RCTs on Nimodipine included 1,157 patients and 1,138 controls. Eight RCTs on Memantine comprised 545 patients and 542 controls. Furthermore, seven RCTs on Rivastigmine included 558 patients and 598 controls. Thirty-one RCTs on Butylphthalide involved 1,396 patients and 1,398 controls. Five RCTs on Cerebrolysin encompassed 212 patients and 246 controls. Sixteen RCTs on Huperzine A comprised 572 patients and 542 controls. Four RCTs on Tongxinluo capsule involved 177 patients and 176 controls. Five RCTs on Edaravone included 192 patients and 190 controls. Fourteen RCTs on Tianzhi granule included 914 patients and 868 controls. Among these studies, 186 were two-arm trials, five were three-arm trials, and three were multi-arm trials. The median follow-up duration ranged from 2 weeks to 22 months. Female participants constituted approximately 48.28% of the total sample. The detailed process of data extraction from the included randomised controlled trials is summarised in Supplementary Table 1 (L. J, 2018; JC, 2014; L. M, 2016; Marigliano et al., 1992; Wang G et al., 2020; Wang GF, 2015; Wu XH et al., 2020; Zhang DP et al., 2013; Zhang FZ et al., 2015; Bottini et al., 1992; Cai QL and Jiang, 2017; Cheng QS and Zhou, 2014; Dysken et al., 1989; GJ, 2011; Haili Binuer, 2003; HF, 2006; Huang, 2012; Li M and Bo, 2010; Liu L and Zhao, 2015a), (Liu L and Zhao, 2015a; Lu Y, 2011; Y. M, 2013; Maina et al., 1989; Ni YY and Tang, 2016; R, 2021; RQ, 2013; S, 2021; SW, 2016; SY, 2019; TW, 2017; Villardita et al., 1992; XB, 2016; Z. Y, 2009; YD, 2013; Yu WY and Zhao, 2008; Yue YM and Zhang, 2013; Zhong H and Li, 2014; Auchus et al., 2007; Erkinjuntti et al., 2002; RP, 2014; Ballard et al., 2008; Mok et al., 2007; Moretti et al., 2002; Moretti et al., 2003; Moretti et al., 2004; Xiao J et al., 2004; Zhong XD, 2021; Guekht et al., 2011; X. H, 2016; Muresanu et al., 2008; Muresanu et al., 2010; Wang J, 2016; Chen H, 2012; FC, 2007; FJ, 2010; HH, 2009; Lu JH et al., 1998; Song YJ and Li, 2006; SY, 2013; Zhang YH and Geng, 2008; Chen L and Yang, 2013; Roman et al., 2010a) (Pantoni et al., 2005; Pantoni et al., 2000; SF, 2012; Sui CF and Wang, 2001; Wang YF, 2009; Wang ZF, 2016; Wang ZG et al., 2009; XH, 2016; Xu XY and Huang, 2017; XY, 2009; XZ, 2018; Zhou Y et al., 2021; W. F, 2017; H, 2015; Liu L and Zhao, 2015b; Wang FL, 2011; Wang RP and Hu, 2004; Wang WL et al., 2010; Weng QL et al., 2011; Xu ZJ et al., 2009; Xu et al., 2012; Xu ZQ et al., 2009; YQ, 2000; Zhagn, 2013; Zhang SG, 2011; Zhao M and Zhang, 2008; Zheng GL and Li, 2006; Zhong, 2004; Du JG et al., 2003; GL, 2016; JT, 2011; Lian CL et al., 2010; Liu XP, 2009; QY, 2009; SH, 2012; Shi et al., 2020; SL, 2011), (Orgogozo et al., 2002; Ouyang XC et al., 2010; Wilcock et al., 2002; Yao MR et al., 2015; Du W, 2012; HQ, 2012; Li HY and Zhu, 2010; Zhang F, 2010; Zhou GA and Ning, 2012; Chang LX and Cai, 2019; Cheng WT, 2016; DY, 2013; FQ, 2015; GF, 2012; H. H, 2017; He, 2016; Hou et al., 2009; Li, 2018; Liu C and Zheng, 2013; Liu J and Liu, 2020; Long CY et al., 2012; Lu HL et al., 2011; Lv Cuilan and Feng, 2015; Ma J, 2018; L. S, 2015), (L. S, 2015; W. S, 2021; Shao XP and Hu, 2013; Sheng GL, 2015; Su XD, 2015; Sun et al., 2016; Wang QY et al., 2014; Wang YJ and Tu, 2013; Wu J et al., 2012; Xiao, 2019; XL, 2015; Xu F et al., 2015; XY, 2019; Y. Y, 2020; YB, 2018; Black et al., 2003; BO Yu-qing, 2012; Chen GQ, 2018; Chen SQ et al., 2007; CY, 2012; P. D, 2014; Dichgans et al., 2008; DR, 2013; Du CB, 2022; Roman et al., 2010b; HM, 2010), (Kavirajan and Schneider, 2007; Kong YN and Chen, 2007; L. L, 2009; Li G et al., 2019; Li N, 2005; Lin JY et al., 2007; W. LL, 2022; Pratt and Perdomo, 2002; Qiu YH et al., 2011; Ren XY et al., 2004; Román et al., 2010; Román et al., 2005; Rong JC, 2011; Schindler, 2005; Tan AX, 2018; Wang Y et al., 2006; Wen K, 2006; Wilkinson et al., 2003; Wilkinson et al., 2010; H. WL, 2010; WX, 2014; XL, 2016; Q. Y, 2017; YF, 2018; YW, 2010)

### Risk-of-bias assessment

**FIGURE 2:**
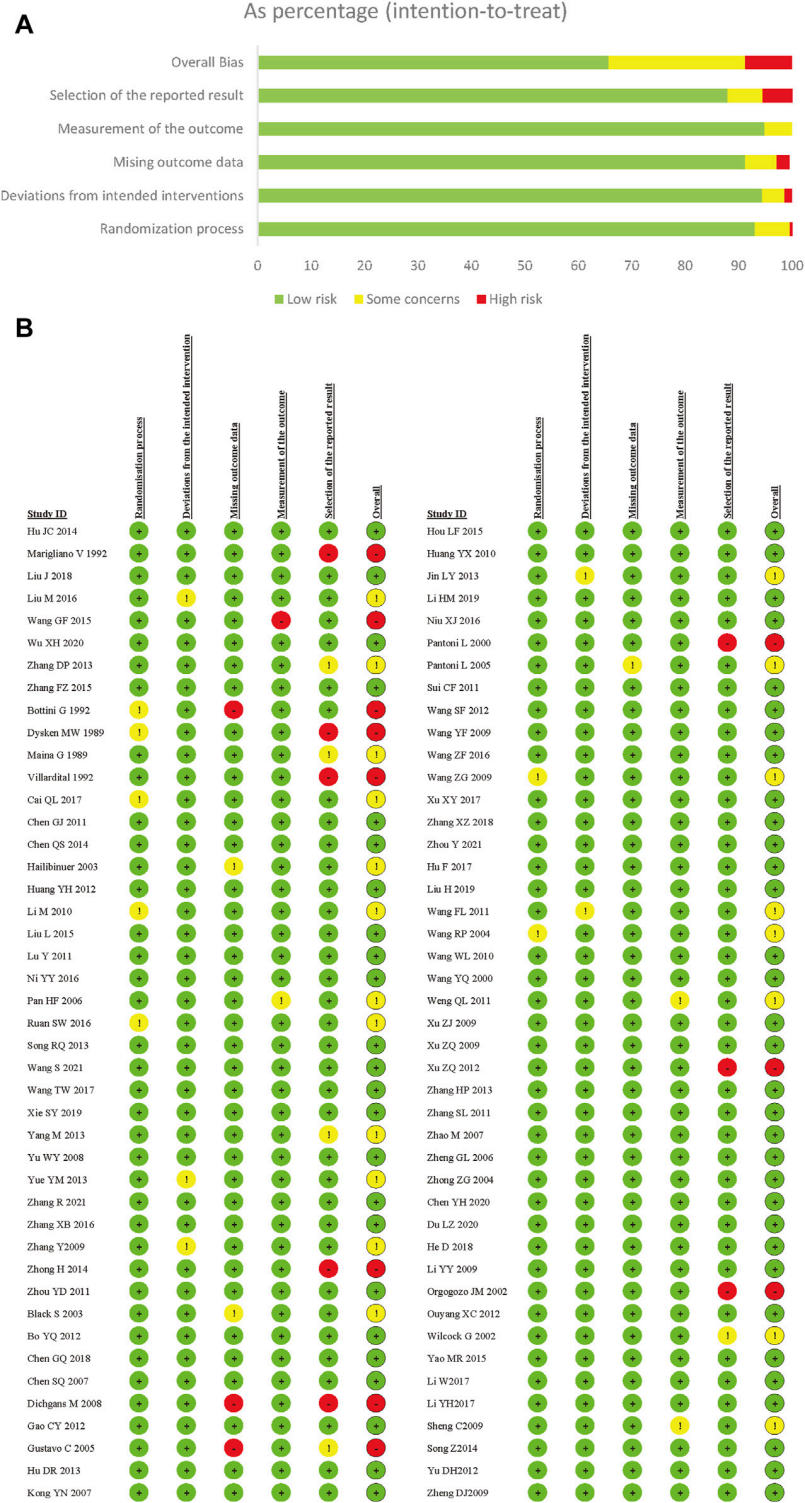
Risk of bias summery for vascular dementia. **(A)** Risk of bias graph for vascular dementia; **(B)** Risk of bias summary for vascular dementia.

In this NMA review, all included trials underwent a risk of bias assessment using the Risk of Bias 2.0 Tool. A major factor contributing to high bias risk was the selection of the reported results. Due to inadequate details regarding allocation concealment, there were general or substantial concerns about the randomization process. Out of all the trials, 124 (63.9%) were evaluated as low risk, 50 (25.8%) were deemed moderate risk, and 20 (10.3%) were categorized as high risk. Collectively, the studies presented a low to moderate risk of bias (Figure 2).

### Heterogeneity and consistency test

**FIGURE 3:**
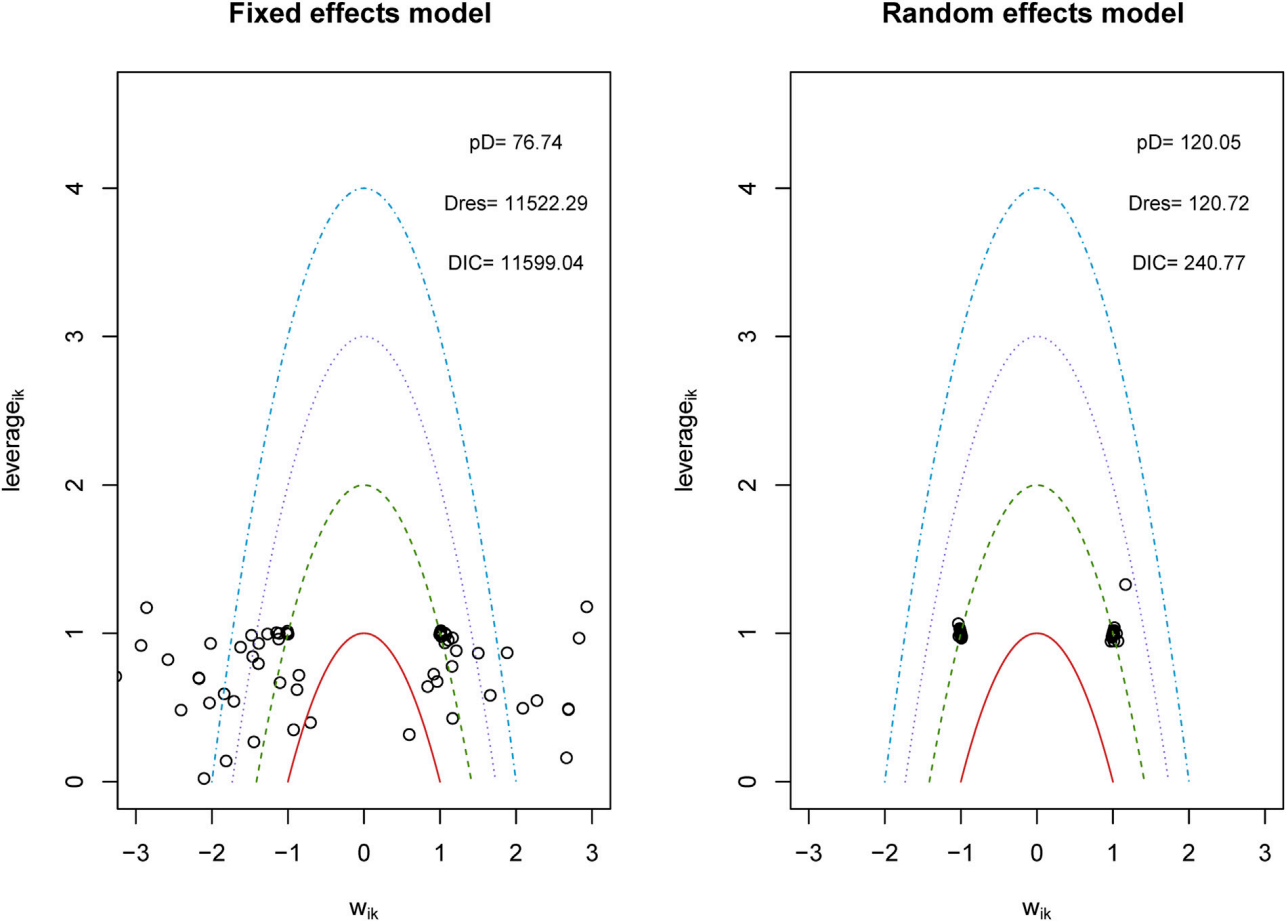
Conformance test for vascular dementia. Conformance test compares the posterior mean deviation of each data group between consistency and the ume m(b) Bias risk evaluation results displayed by including studies odel to judge the consistency among the included research.

**FIGURE 4:**
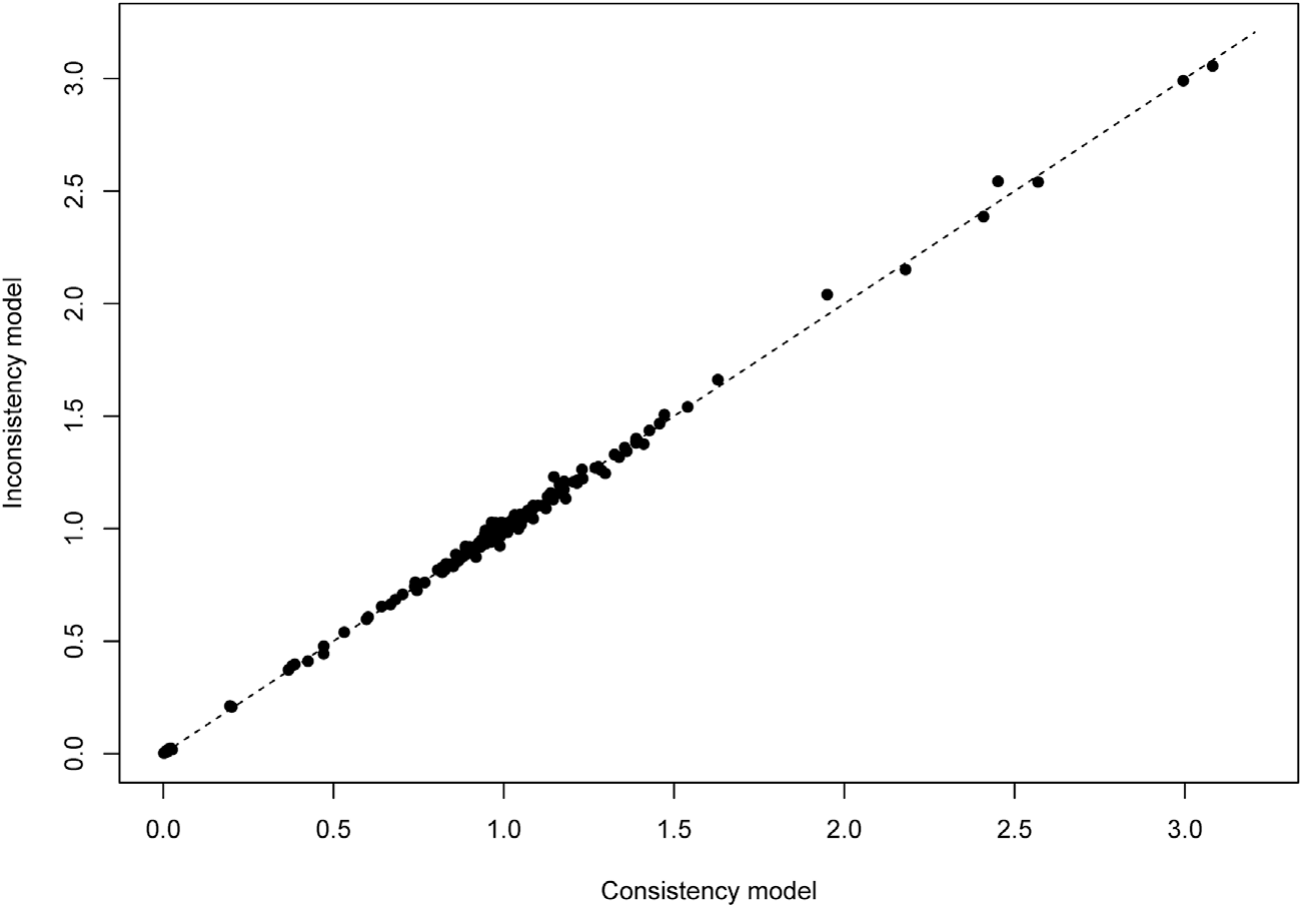
Lever diagram for vascular dementia. The lever diagram shows the comparison between leverageik and Bayesian deviation residuals of all I tests and each of the K arms.

**FIGURE 5:**
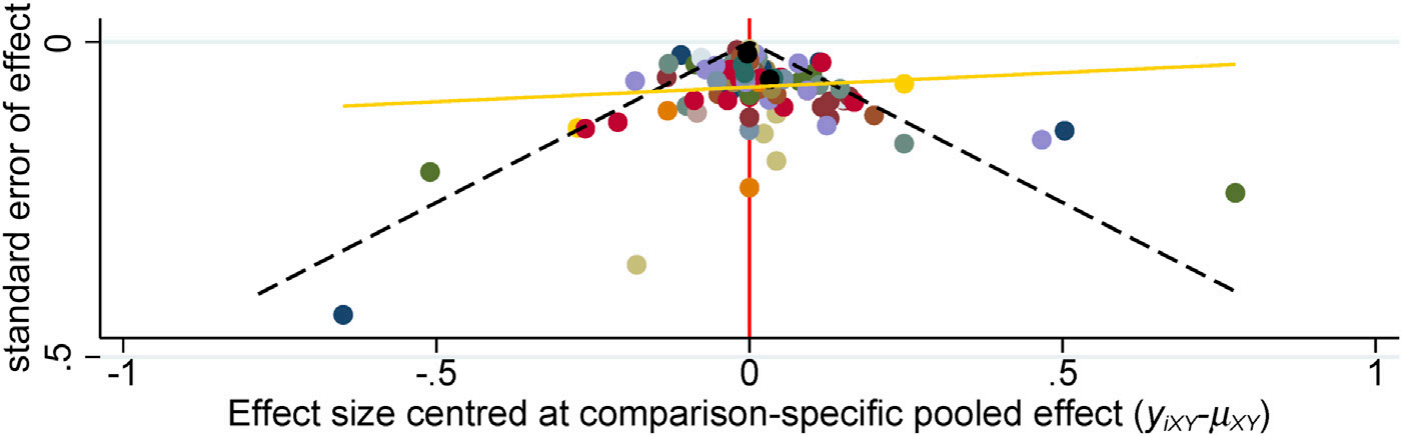
Funnel plots for systematic review and meta-analysis.

We analyzed the model fit between the fixed-effects and random-effects models for various outcomes, as shown in Figure 3, and evaluated model consistency by analyzing the posterior distribution of deviance differences, with results presented in Figure 4. Our findings indicate a superior fit of the random-effects model across all outcomes, affirming the consistency and validity of indirect comparisons. Funnel plots, depicted in Figure 5, suggest minimal publication bias due to their symmetrical distribution.

Trajectory plots in Supplementary Figure 1A illustrate the parameter estimates’ evolution and stabilization, key indicators of model convergence, demonstrating stable fluctuations and significant overlap in the MCMC chain. Density plots, shown in Supplementary Figure 1B, display parameter distributions and suggest excellent model convergence through symmetrical and well-calibrated curves. The Brooks-Gelman-Rubin diagnostic diagram in Supplementary Figure 2 assesses the convergence of multiple chains, with values nearing one indicating robust convergence.

Overall, the consistent convergence patterns in the trajectory and density plots, alongside the Brooks-Gelman-Rubin diagnostic, confirm the robustness and reliability of the model’s predictions.

### Network diagram

**FIGURE 6:**
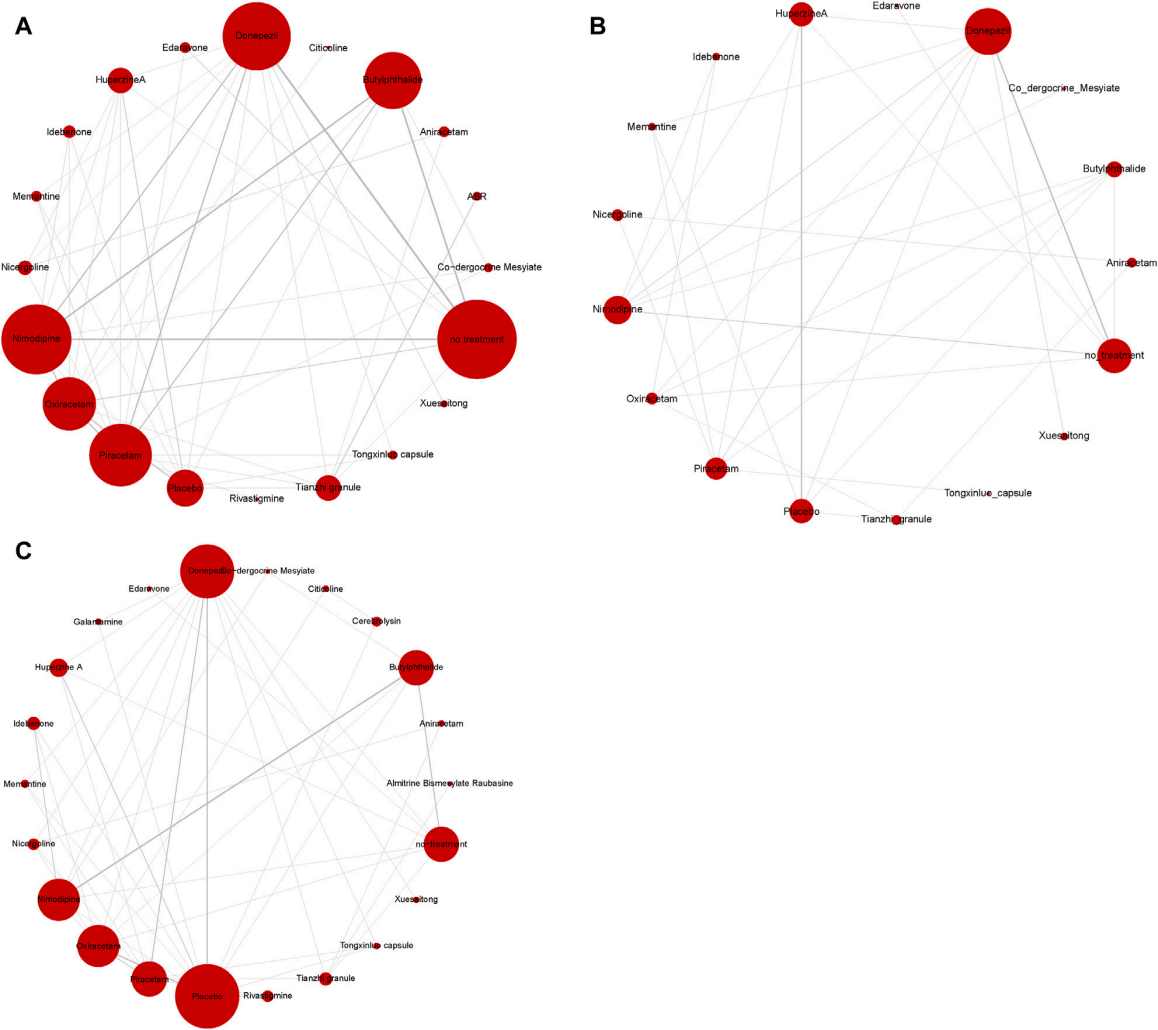
Network diagram of available comparisons. **(A)** MMSE scores; **(B)** ADL scores; **(C)** adverse effects rate.

The process of creating a network diagram in R includes installing the required packages, preparing the data, constructing the network object, plotting the diagram, and refining its aesthetics. This function generates network diagrams for outcome indicators as required. These diagrams feature drug nodes, with node sizes corresponding to the number of participants randomly assigned (i.e., sample size), and comparison edges, where the thickness of the lines indicates the number of trials comparing each pair of treatments. A closed loop among nodes signifies that these studies can be compared simultaneously. The results showed that the number of studies comparing no treatment with Donepezil was the largest, followed by comparing no treatment with Nimodipine (Figure 6).

### Forest plot

**FIGURE 7:**
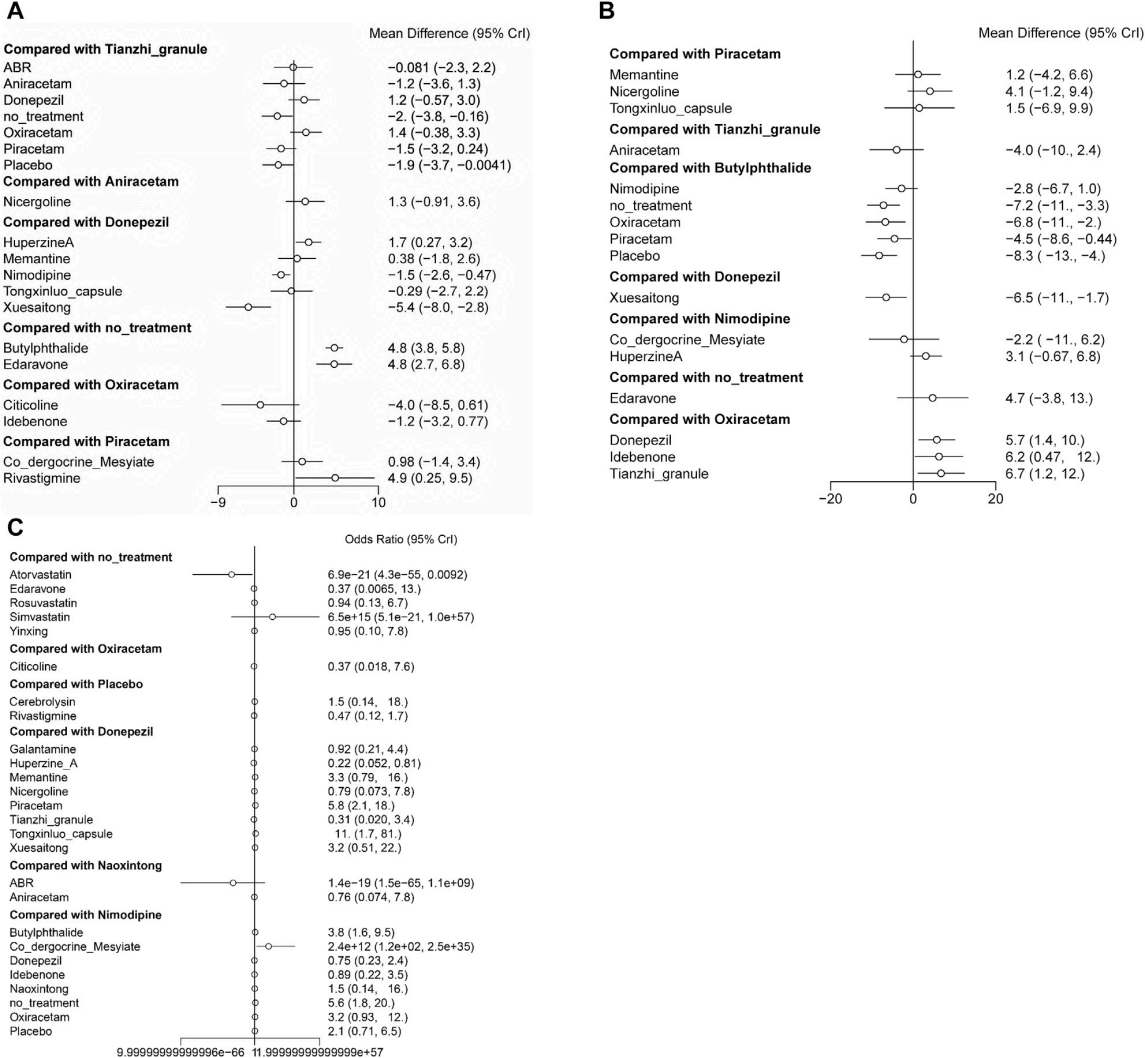
Forest plots representing the efficacy and safety of patients with vascular dementia. **(A)** direct comparison for MMSE scores; **(B)** direct comparison for ADL scores; **(C)** direct comparison for adverse effects rate.

In terms of MMSE scores, Huperzine A demonstrates superior efficacy compared to Donepezil, while Nimodipine and Xuesaitong exhibits inferior efficacy. Both Edaravone and Butylphthalide show greater efficacy relative to no treatment. Rivastigmine is more effective than Piracetam (Figure 7A).

Regarding ADL scores, Oxiracetam and Piracetam are less effective compared to Butylphthalide, and Xuesaitong is less effective than Donepezil. Conversely, Donepezil, Idebenone, and Tianzhi granule display higher efficacy compared to Oxiracetam (Figure 7B).

In terms of adverse reaction incidence, Co-dergocrine mesylate is safer than Nimodipine, whereas Atorvastatin presents a higher risk compared to no treatment. The remaining data did not show statistical significance (Figure 7C).

### Heat map

In NMA, a heat map is an informative graphical representation that illustrates the distribution and strength of evidence across various treatment comparisons. Each row and column of the heat map corresponds to different treatments, with cells indicating the presence of direct comparative data between treatments.

**FIGURE 8:**
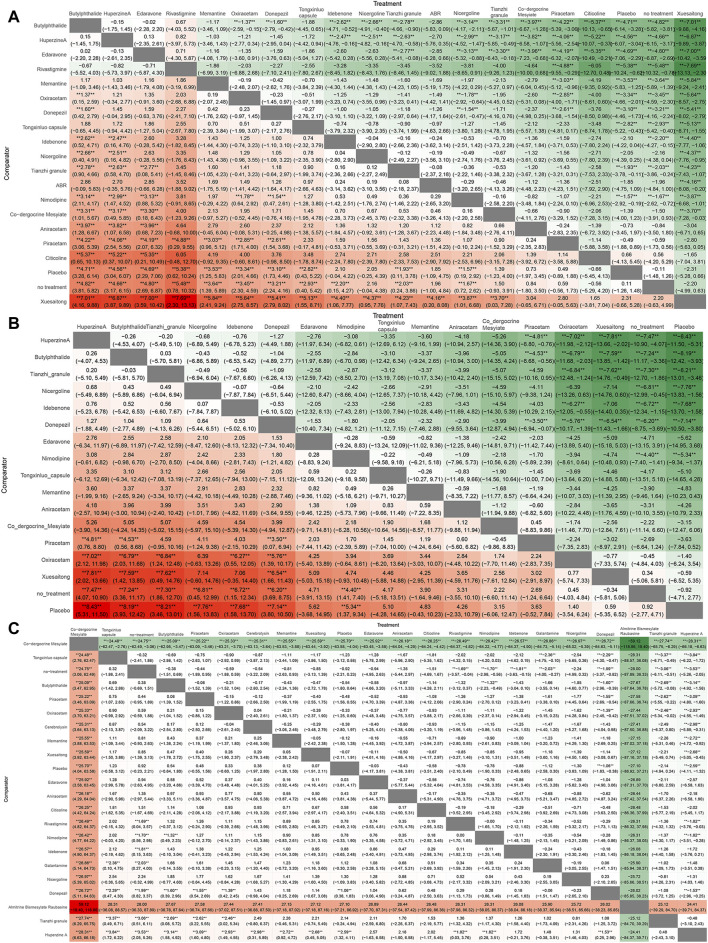
Ranking chart heat map. The heat map of each outcome index ranking table presented comparisons of the relative effects between any pair of interventions, including the OR and 95%CI of each outcome index in all intervention groups. **(A)** MMSE scores; **(B)** ADL scores; **(C)** adverse effects rate.

The comparative results of the various drugs are illustrated in Figure 8. Butylphthalide exhibited superior efficacy in terms of changes in MMSE scores, surpassing Oxiracetam, Donepezil, Idebenone, Nicergoline, Nimodipine, Co-dergocrine Mesylate, Aniracetam, Piracetam, Citicoline, and Xuesaitong (Figure 8A). Huperzine A demonstrated superior effectiveness in improving ADL scores compared to other treatments, including Piracetam, Oxiracetam, Xuesaitong, and Placebo (Figure 8B). Co-dergocrine Mesylate showed a better safety profile in terms of the incidence of adverse reactions, outperforming Tongxinluo capsule, Butylphthalide, Piracetam, Oxiracetam, Cerebrolysin, Memantine, Xuesaitong, Edaravone, Aniracetam, Citicoline, Rivastigmine, Nimodipine, Idebenone, Galantamine, Nicergoline, Donepezil, Tianzhi granule, and Huperzine A (Figure 8C).

### SUCRA rankings, cumulative probability ranking chart and ranking probability histogram

SUCRA is a statistical method used in NMA to quantify the probability of each treatment being the most effective among all compared treatments. It provides a numerical value between 0 and one for each treatment, where a higher SUCRA value indicates a higher likelihood of that treatment being the best (Table 1). The SUCRA and rankogram charts intuitively display the sorting probability of each intervention group in the form of curves and histogram.

**TABLE 1:** SUCRA rankings.

Efficacy			safety		
MMSE	SUCRA	ADL	SUCRA	adverse effects rate	SUCRA
Butylphthalide	90.32	HuperzineA	82.15	Co-dergocrine Mesyiate	99.61
HuperzineA	88.11	Butylphthalide	79.62	Tongxinluo capsule	82.07
Edaravone	87.78	Tianzhi_granule	78.25	no-treatment	80.24
Rivastigmine	86.54	Nicergoline	74.29	Butylphthalide	71.35
Memantine	72.91	Idebenone	73.34	Piracetam	70.49
Oxiracetam	71.31	Donepezil	70.53	Oxiracetam	66.32
Donepezil	67.08	Edaravone	55.52	Cerebrolysin	64.55
Tongxinluo capsule	61.71	Nimodipine	52.33	Memantine	60.1
Idebenone	49.83	Tongxinluo_capsule	51.38	Xuesaitong	58.49
Nicergoline	49.51	Memantine	48.96	Placebo	57.08
Tianzhi granule	47.27	Aniracetam	43.43	Edaravone	53.99
ABR	46.06	Co_dergocrine_Mesyiate	37.74	Aniracetam	48.36
Nimodipine	40.66	Piracetam	37.39	Citicoline	43.73
Co-dergocrine Mesyiate	38.52	Oxiracetam	20.82	Rivastigmine	37.01
Aniracetam	27.99	Xuesaitong	16.9	Nimodipine	36.4
Piracetam	23.08	no_treatment	16.65	Idebenone	34.88
Citicoline	18.41	Placebo	10.71	Galantamine	29.84
Placebo	15.91			Nicergoline	29.33
no treatment	14.07			Donepezil	29.01
Xuesaitong	2.86			ABR	21.76
				Tianzhi granule	16.82
				Huperzine A	8.62

*MMSE: Mini-Mental State Examination; ADL: activities of daily living; ABR: almitrine bismesylate raubasine.

**FIGURE 9:**
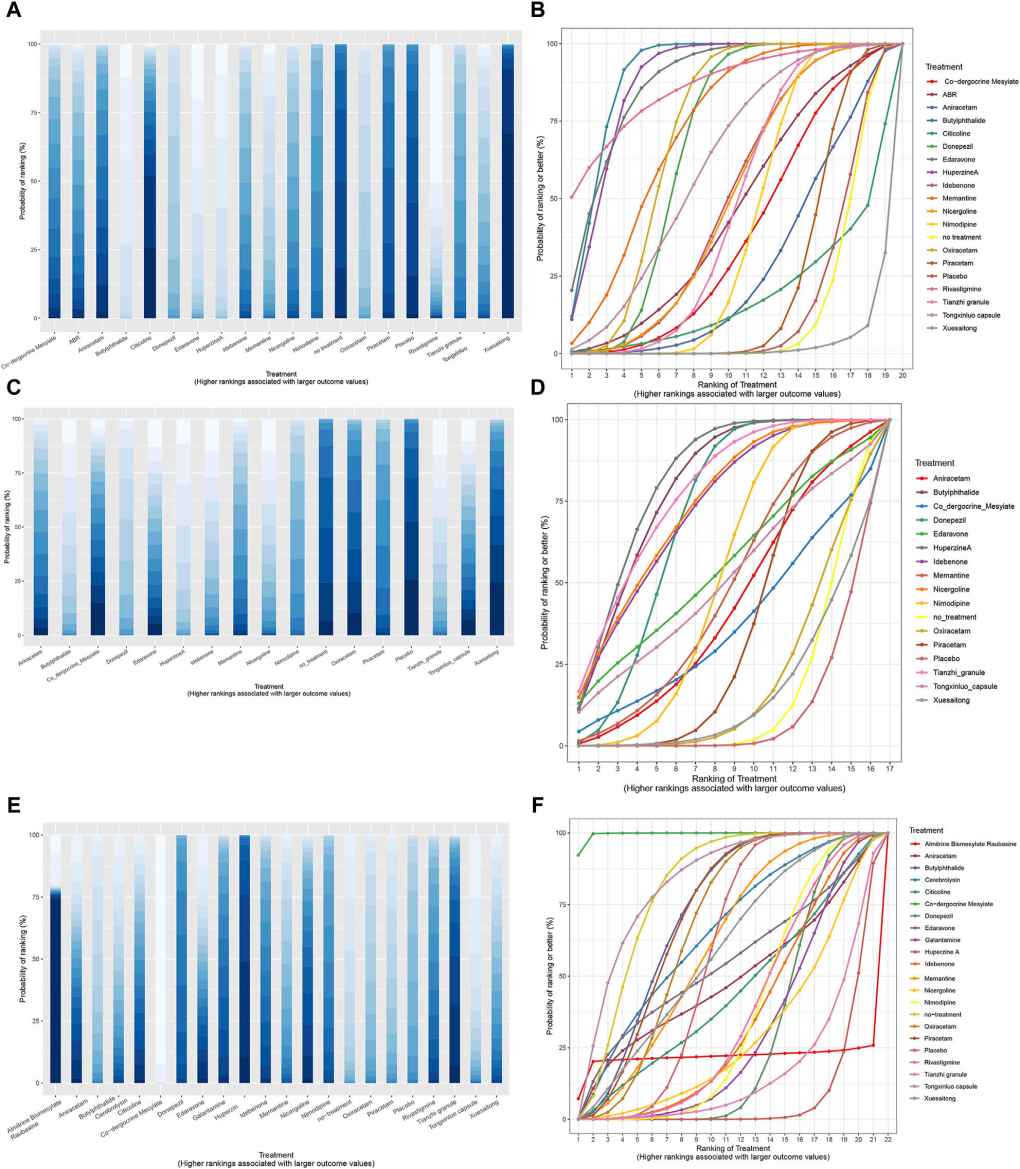
SSUCRA cumulative probability ranking curves and histogram. **(A)** histogram of MMSE scores; **(B)** SUCRA curve chart of MMSE scores; **(C)** histogram of ADL scores; **(D)** SUCRA curve chart of ADL scores; **(E)** histogram of adverse effects rate; **(F)** SUCRA curve chart of adverse effects rate.

In terms of MMSE scores, the five most effective drugs are Butylphthalide, Huperzine A, Edaravone, Rivastigmine, and Memantine. For ADL scores, the top five drugs in efficacy are Huperzine A, Butylphthalide, Tianzhi granule, Nicergoline, and Idebenone. With respect to the incidence of adverse drug reactions, Co-dergocrine Mesylate, Tongxinluo capsule, Butylphthalide, Piracetam, and Oxiracetam demonstrate good safety profiles (Figure 9).

## Discussion

This study is the first Bayesian NMA to evaluate the efficacy and safety of various treatments for VaD. We performed an exhaustive search and assessed crucial outcomes including the MMSE score, ADL score, and the incidence of adverse events. These outcomes were analyzed through direct and indirect comparisons, enhancing the evidence’s reliability. The included studies were generally found to have low-to-moderate risk of bias, as assessed by the Cochrane Risk of Bias 2.0 Tool, affirming the robustness of our findings.

### Key findings

This study provides a comprehensive analysis of various pharmacological treatments for VaD using the MMSE scale to reflect cognitive state, the ADL scale to measure daily living activities, and the adverse reaction rate as a safety indicator. The NMA reveals several key findings regarding the efficacy and safety profiles of these drugs.

Huperzine A demonstrates good efficacy in both MMSE and ADL scores but has the poorest safety profile. Butylphthalide shows robust efficacy in both scales and a low adverse reaction rate, indicating it is both effective and safe. Edaravone ranks high in MMSE scores, mid-range in ADL scores, and has a mid-range safety profile. Memantine and Oxiracetam perform well in MMSE scores but rank lower in ADL scores, though both have high safety rankings. Donepezil is effective in improving MMSE and ADL scores but is associated with poor safety. On the other hand, Tongxinluo capsule and Co-dergocrine Mesylate are noted for their excellent safety profiles but rank poorly in terms of efficacy. Rivastigmine shows high efficacy in MMSE scores but also has poor safety. These findings underscore the importance of considering both efficacy and safety when selecting pharmacological treatments for VaD. While some drugs, such as Butylphthalide, offer a balanced profile of high efficacy and low risk of adverse reactions, others like Huperzine A and Donepezil, despite their effectiveness, pose significant safety concerns. Thus, a nuanced approach that balances these factors is essential for optimizing treatment outcomes in VaD patients.

### Drug mechanism

Current therapeutic approaches for VaD primarily focus on modulation of the cholinergic system, neuroprotection, and the use of anti-inflammatory and antioxidant agents (Colucci et al., 2012; McShane et al., 2019).

Cholinesterase inhibitors such as donepezil, galantamine, and rivastigmine enhance cognitive function by increasing intrasynaptic acetylcholine levels, a neurotransmitter integral to memory and learning processes (Román and Kalaria, 2006; Chen et al., 2016). Each of these inhibitors has distinct structural, pharmacodynamic, and pharmacokinetic properties. For instance, donepezil is characterized by a long elimination half-life, galantamine modulates nicotinic receptor function, and rivastigmine also inhibits butyrylcholinesterase, which contributes to its efficacy in treating VaD (Cummings, 2000; Kavirajan and Schneider, 2007). Nicergoline, a semisynthetic ergot derivative, has been used extensively for over 4 decades to treat cognitive and behavioural disorders in the elderly (Fioravanti et al., 2014). It enhances acetylcholine availability by increasing its release from cholinergic terminals and selectively inhibiting acetylcholinesterase. Furthermore, nicergoline stimulates the phosphoinositide pathway, affecting neurotransmitter turnover including norepinephrine and dopamine in specific brain regions (Carfagna et al., 1995; Winblad et al., 2008). Additionally, huperzine A, an alkaloid derived from the Chinese herb Huperzia serrata, acts as a potent, highly specific, and reversible inhibitor of acetylcholinesterase in the central nervous system. Since 1994, it has been used in China to treat AD and benign memory deficits and is available as a nutraceutical in the US (Little et al., 2008). Oxiracetam, another nootropic, is employed with the aim of improving executive function and memory (Malík and Tlustoš, 2022). It readily crosses the blood-brain barrier, affecting areas such as the cortex and hippocampus. It enhances acetylcholine utilization and increases the density of acetylcholine receptors by activating cholinergic nerve fibers, and it has been shown to normalize brain energy balance and alleviate blood-brain barrier dysfunction during brain ischemia in experimental models (Hokonohara et al., 1992; Fordyce et al., 1995; Huang et al., 2017; Youn et al., 2023). Memantine, a well-known uncompetitive and voltage-dependent NMDA receptor antagonist, mitigates the excitotoxicity caused by excessive ischemia-induced NMDA receptor stimulation, offering protection against cognitive decline in VaD (Koch et al., 2005; Burns et al., 2006; Johnson and Kotermanski, 2006). These multifaceted effects underscore the complexity and potential of current pharmacological interventions for VaD.

Accumulating evidence increasingly highlights the critical roles of oxidative stress and inflammation as key factors contributing to the cognitive deficits observed in patients with VaD. Current research indicates that the induction of oxidative stress and inflammatory cascades can lead to a decline in NMDA receptor functionality. This decline may result in subsequent alterations in synaptic plasticity, ultimately leading to cognitive impairment (Wang et al., 2020; Guo et al., 2024). Butylphthalide, derived from *Apium graveolens* Linn and also synthesized artificially from racemic acid, is a green botanical medicine approved by China Food and Drug Administration (CFDA) for the treatment of ischemic stroke due to its neuroprotective properties. Recent RCTs have demonstrated that butylphthalide can enhance behavioral abilities and alleviate symptoms of VaD. Its therapeutic effects are potentially mediated by the upregulation of phosphorylated Akt in the hippocampus, improves mitochondrial function, contributing to its cognitive benefits (Huai et al., 2013; Liu et al., 2024; Zhang et al., 2024). Additionally, idebenone, recognized for its potent antioxidant properties and as a CoQ10 analogue, was initially developed to combat dementia. It plays a crucial role in the clinical treatment of cerebrovascular diseases, helping to eliminate oxygen free radicals, counteract oxidation, enhance mitochondrial respiratory activity, and improve overall brain function and metabolic status (Marigliano et al., 1992). Edaravone, chemically known as 3-methyl-1-phenyl-2-pyrazoline-5-one, is a nootropic and neuroprotective agent that aids neurological recovery following acute brain ischemia and subsequent cerebral infarction. First approved for clinical use in Japan in 2001, Edaravone potentially upregulates the activity of Akt, alleviating oxidative stress, restores synaptic proteins, and improves memory deficits, thus playing a crucial role in the treatment of neurodegenerative conditions (Yoshida et al., 2006; Li et al., 2017). Additionally, naoxintong capsule, a composite formulation of 16 natural ingredients, including 13 plant-based and three animal-based components has been extensively utilized in China for treating VaD. Additionally, tongxinluo capsule, containing components like dehydroevodiamine and evodiamine, potentially blocks glutamatergic signaling and reduces inflammation, contributing to its therapeutic effects (Chan et al., 2018). Tianzhi granule, a traditional herbal medication, has been approved by CFDA for treating VaD. Comprising a blend of various herbs including Gastrodia elata, Uncaria rhynchophylla, Abalone shell, Eucommia ulmoides, Mulberry mistletoe, Poria cocos, Polygonum multiflorum, Sophora japonica, Gardenia jasminoides, Scutellaria baicalensis, Achyranthes bidentata, and Leonurus japonicus, tianzhi granule inhibits the proliferation of astroglial cells while promoting the proliferation of precursor nerve cells, thus enhancing learning and memory capabilities in VaD rat models (Zhao et al., 2022). Morover, cerebrolysin, a peptidergic compound that mimics the action of endogenous neurotrophic factors, addresses neuroinflammation and oxidative stress. Due to its composition, cerebrolysin requires parenteral administration to achieve full bioavailability and is typically administered over a short period due to its mode of action. It penetrates the blood-brain barrier to regulate the synthesis of proteins and metabolism of nucleic acids in brain cells, enhancing the utilization of oxygen and glucose, and offering resistance to neurotoxic substances. However, evidence on the cognitive efficacy of cerebrolysin remains limited (Bae et al., 2000; Ruether et al., 2001; Ruether et al., 2002; Rockenstein et al., 2005; Allegri and Guekht, 2012). These findings emphasize the need to address these underlying biological processes to mitigate their impact on cognitive function in VaD.

Vascular risk factors such as hypertension, diabetes, smoking, elevated cholesterol, and atherosclerosis are critical in the development of cerebrovascular disease, leading to vascular brain injury and subsequent vascular cognitive impairment and VaD. The primary strategy for managing VaD involves targeting these modifiable risk factors, either to prevent or delay disease progression or to alleviate associated behavioral symptoms, including secondary stroke prevention (Takeda et al., 2020). Early detection and mitigation of these vascular risk factors are essential for the effective prevention and treatment of vascular cognitive impairment and dementia. Nimodipine, a 1,4-dihydropyridine-derivative calcium channel blocker, exhibits significant antihypertensive properties and a unique cerebrovascular profile. Due to its high lipophilicity, nimodipine can cross the blood-brain barrier, effectively reaching the brain and cerebrospinal fluid. Clinical studies generally show favorable effects of nimodipine in hypertensive patients, including reductions in systolic and diastolic blood pressure, improvements in subjective symptoms, and neurological enhancements that contribute to improved cognitive function (Tomassoni et al., 2008; Amenta et al., 2009). However, the specific mechanisms underlying the neuroprotective effects of nimodipine remain partly unclear (Baskys and Hou, 2007).

### Strengths

In contrast to previous reviews and meta-analysis, this NMA firstly performs a graded quantitative analysis of the efficacy and safety of all commonly used drugs in patients with VaD. The use of Bayesian NMA allowed for a robust comparison of multiple treatments, integrating direct and indirect evidence to provide a comprehensive ranking of their effectiveness and safety. This methodology enhances the reliability of the findings and supports evidence-based clinical decision-making. By integrating direct and indirect evidence in our study, we provide clinicians with the most current evidence to inform them when new therapies might be applicable. To enhance the rigor and evidence-based strength of our findings, this study exclusively included interventional clinical trials, adhering to stringent inclusion/exclusion criteria to ensure that the trials incorporated are not only the most recent and comprehensive but also of the highest quality. Unlike previous meta-analysis, which primarily focused on cholinesterase inhibitors, this innovative network analysis encompasses nearly all current medications for VaD, including Western medicines and traditional Chinese medicines. Importantly, the potential benefits of traditional Chinese herbal medicine in treating VaD have been widely explored, positioning these as alternative therapeutic options. Consequently, this study is more comprehensive and covers a broader spectrum of medications than previous studies. The study underscores the potential of several pharmacological agents, including traditional Chinese medicines, in treating VaD. However, the varying levels of efficacy and safety across different treatments highlight the need for personalized treatment approaches and further research to establish standardized therapeutic guidelines.

### Limitation

Our study has several limitations that warrant consideration. Firstly, variability in drug dosages and treatment durations across the included RCTs may have influenced outcomes. Secondly, the specific characteristics of patient populations, such as the severity of VaD, age, and gender, could affect the effectiveness and safety of the treatments evaluated. Thirdly, the inclusion of numerous studies with small sample sizes restricts the certainty of the evidence for clinical application. Fourthly, while we used the MMSE and ADL scores as primary efficacy outcomes, other VaD scales like the Blessed-dementia rating scale, Hasegawa dementia scale, and AD Assessment Scale-cognitive subscale were excluded due to insufficient data from clinical trials. This exclusion might limit broader conclusions about the efficacy of treatments, particularly Chinese herbal medicines. Finally, the follow-up duration in the included trials was approximately 22 months, which may be too brief to fully assess the long-term effectiveness of the treatments given the typically gradual progression of the disease.

## Conclusion

Overall, this study enhances understanding of the relative benefits and risks associated with various VaD treatments, providing a valuable reference for clinical decision-making. Future research should continue to explore these aspects to further refine treatment strategies for VaD.

## Data Availability

The raw data supporting the conclusions of this article will be made available by the authors, without undue reservation.
