# Supplementary figures and images for: Pharmacological treatments for vascular dementia: a systematic review and Bayesian network meta-analysis
Source: Front Pharmacol. 2024 Aug 22;15:1451032. doi: 10.3389/fphar.2024.1451032 (PMC11374729; doi:10.3389/fphar.2024.1451032)

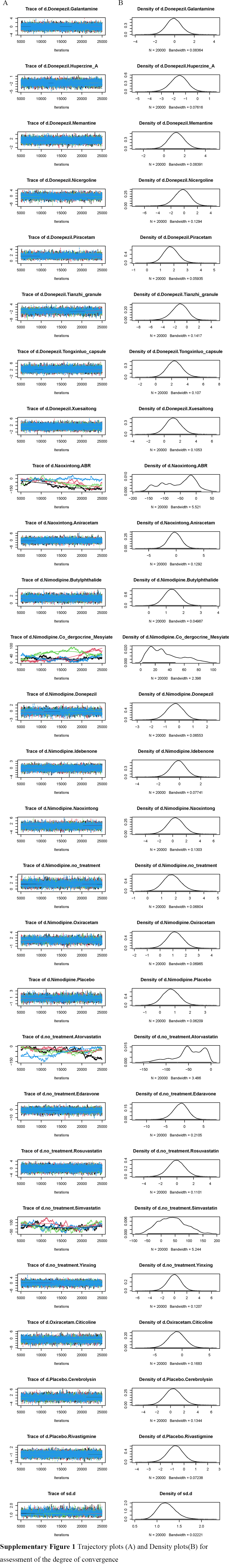

Supplement: Supplementary file 1 [file Image1.TIFF]

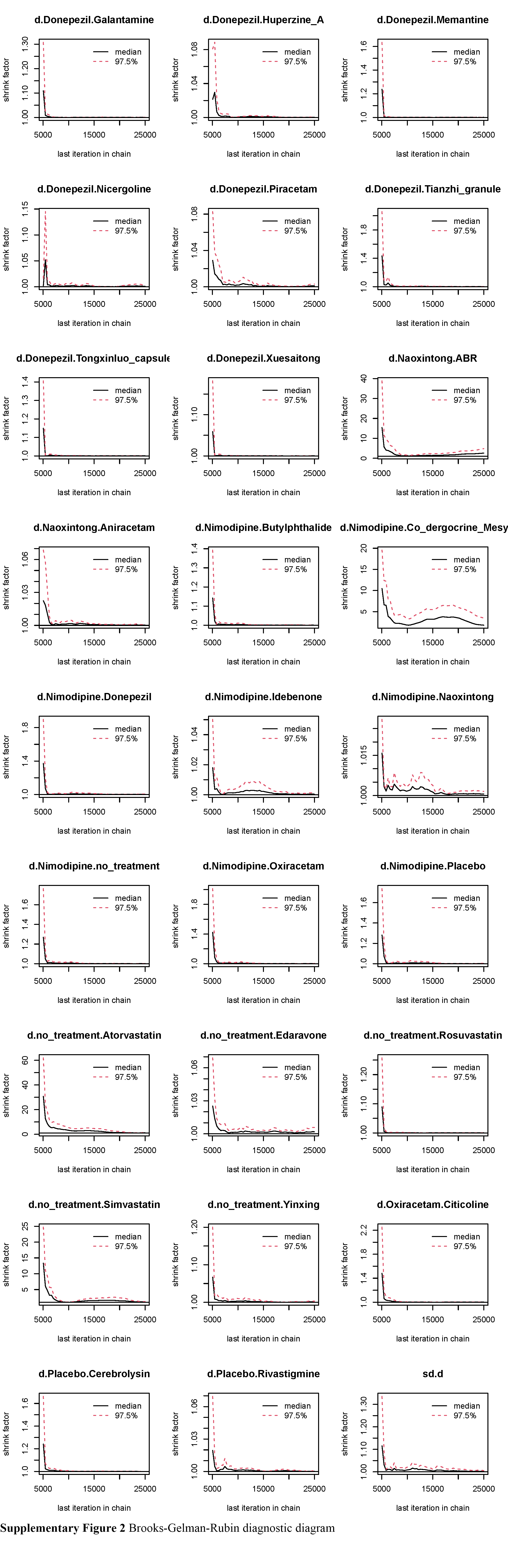

Supplement: Supplementary file 3 [file Image2.TIFF]
