# Supplementary material for: Pharmacological treatments for vascular dementia: a systematic review and Bayesian network meta-analysis
Source: Front Pharmacol. 2024 Aug 22;15:1451032. doi: 10.3389/fphar.2024.1451032 (PMC11374729; doi:10.3389/fphar.2024.1451032)
Supplement: Supplementary file 2 [file Table1.docx]

| **Supplementary Table 1** Basic characteristics of the included studies | | | | | | | | | | | | | | |
| --- | --- | --- | --- | --- | --- | --- | --- | --- | --- | --- | --- | --- | --- | --- |
| **Study** | **Patients of IG** | **Male** | **Female** | **Patients of CG** | **Male** | **Female** | **Age of IG, CG(mean±SD)** | **Age of CG, CG(mean±SD)** | **Treatment intervention** | **Interventions dose route** | **Control intervention** | **Control measures dose route** | **Outcome measure** | **Follpw-up（week）** |
| Hu JC2014 | 47 | 22 | 25 | 45 | 20 | 25 | 69.5±7.6 | 70.4±6.9 | Idebenone | po, tid, 40mg | Nimodipine | po, tid, 40mg | ③ | 8 |
| Liu J2018 | 39 | 21 | 18 | 39 | 22 | 17 | 64.8±4.2 | 65.0±4.3 | Idebenone | po, tid, 40mg | Nimodipine | po, tid, 30mg | ③ | 12 |
| Liu M2016 | 51 | 26 | 25 | 51 | 28 | 23 | 63.57±7.09 | 63.28±7.32 | Idebenone | po, tid, 30mg | Nimodipine | po, tid, 40mg | ①②③ | 12 |
| Wang GF2015 | 40 | 24 | 16 | 31 | 17 | 14 | 65.1±5.2 | 66.9±4.9 | Idebenone | po, tid, 30mg | Nimodipine | po, tid, 30mg | ①②③ | 12 |
| Wu XH2020 | 20 | 13 | 7 | 20 | 8 | 12 | 63.4±11.3 | 62.7±11.8 | Idebenone | po, tid, 30mg | Oxiracetam | iv, qd, 4g | ①② | 2 |
| Zhang DP2013 | 31 | 14 | 17 | 26 | 11 | 15 | 64±7 | 65±7 | Idebenone | po, tid, 30mg | Oxiracetam | po, tid, 800mg | ①③ | 12 |
| Zhang FZ2015 | 34 | 16 | 18 | 31 | 14 | 17 | 67±7 | 66±7 | Idebenone | po, tid, 30mg | Nimodipine | po, tid, 40mg | ①③ | 12 |
| Marigliano V1992 | 56 | 28 | 28 | 52 | 25 | 27 | 73.9±4.81 | 73.7±4.74 | Idebenone | po, bid, 20 mg | placebo | NA | ③ | 16 |
| Cai QL2017 | 78 | 48 | 30 | 78 | 44 | 34 | 62.8±2.3 | 65.3±2.8 | Oxiracetam | po, tid, 0.8g | placebo | NA | ①③ | 12 |
| Chen GJ2011 | 53 | 37 | 16 | 53 | 35 | 18 | 65.1 | 65.7 | Oxiracetam | po, tid, 0.8g | no-treatment | NA | ① | 8 |
| Cheng QS2014 | 38 | 20 | 18 | 37 | 22 | 15 | 65.2±8.3 | 66.3±8.4 | Oxiracetam | iv, qd, 6g | Piracetam | iv, qd, 6g | ①③ | 3 |
| Hai2003 | 28 | 19 | 9 | 28 | 18 | 10 | 68±9 | 68±7 | Oxiracetam | po, tid, 0.8g | Piracetam | po, tid, 800mg | ①③ | 8 |
| Huang YH2012 | 27 | 12 | 15 | 29 | 11 | 18 | 71.6±5.1 | 72.2±4.3 | Oxiracetam | po, bid, 1.6g | Donepezil | po, qd, 5mg | ①③ | 12 |
| Li M2010 | 30 | NA | NA | 27 | NA | NA | NA | NA | Oxiracetam | po, tid, 0.8g | placebo | NA | ①③ | 12 |
| Liu L2015 | 22 | NA | NA | 22 | NA | NA | NA | NA | Oxiracetam | po, tid, 0.8g | Huperzine A | po, bid, 0.2mg | ①② | 50 |
| Liu L2015arm2 | NA | NA | NA | 22 | NA | NA | NA | NA | Oxiracetam | po, tid, 0.8g | Nimodipine | po, tid, 40mg | ①② | 50 |
| Lu Y2011 | 80 | 53 | 27 | 80 | 56 | 24 | 69±5.5 | 70±4.9 | Oxiracetam | po, tid, 0.8g | Piracetam | po, tid, 0.8g | ①③ | 12 |
| Ni YY2016 | 60 | 31 | 29 | 60 | 30 | 30 | 63.7±9.3 | 65.1±9.1 | Oxiracetam | iv, qd, 4g | Nimodipine | po, tid, 30mg | ① | 4 |
| Pan HF2006 | 23 | 11 | 12 | 20 | 12 | 8 | 54.2±2.11 | 52.2±2.91 | Oxiracetam | po, tid, 0.8g | Piracetam | po, tid, 0.18g | ①③ | 12 |
| Ruan SW2016 | 48 | 20 | 28 | 48 | 26 | 22 | 55.2±8.3 | 57.2±7.6 | Oxiracetam | iv, qd, 5g | Piracetam | iv, qd, 5g | ①③ | 12 |
| Song RQ2013 | 44 | 30 | 14 | 44 | 28 | 16 | 66.3 | 67.1 | Oxiracetam | po, tid, 0.8g | Piracetam | po, tid, 0.8g | ① | 12 |
| Wang S2021 | 69 | 36 | 33 | 69 | 35 | 34 | 63.38±9.21 | 64.03±9.67 | Oxiracetam | po, tid, 0.8g | Donepezil | po, qd, 5mg | ②③ | 48 |
| Wang TW2017 | 42 | 28 | 14 | 42 | 30 | 12 | 65.44 ±6.30 | 65.50±6.27 | Oxiracetam | iv, qd, 4g | Nimodipine | po, tid, 40mg | ① | 4 |
| Xie SY2019 | 43 | 26 | 17 | 43 | 28 | 15 | 72.46±1.35 | 72.27±1.15 | Oxiracetam | po, tid, 0.8g | placebo | NA | ①③ | 4 |
| Yang M2013 | 34 | NA | NA | 34 | NA | NA | 56.31±11.66 | NA | Oxiracetam | iv, qd, 4g | Nimodipine | po, tid, 40mg | ①③ | 4 |
| Yu WY2008 | 46 | 22 | 24 | 40 | 22 | 18 | 64.2±2.11 | 52.2±2.91 | Oxiracetam | po, tid, 0.8g | Nimodipine | po, tid, 20mg | ① | 24 |
| Yue YM2013 | 49 | 26 | 23 | 49 | 25 | 24 | 53.4±7.5 | 53.5±7.5 | Oxiracetam | iv, qd, 4g | Piracetam | iv, qd, 6g | ①③ | 9 |
| Zhang R2021 | 32 | 18 | 14 | 32 | 19 | 13 | 64.79±3.81 | 64.72±3.84 | Oxiracetam | iv, qd, 4g | no-treatment | NA | ①②③ | 3 |
| Zhang XB2016 | 40 | NA | NA | 40 | NA | NA | 65.3 | 66.2 | Oxiracetam | po, tid, 0.8g | Citicoline | po, bid, 0.2g | ①③ | 16 |
| Zhang Y2009 | 34 | NA | NA | 33 | NA | NA | NA | NA | Oxiracetam | po, tid, 0.8g | placebo | NA | ①③ | 12 |
| Zhong H2014 | 43 | NA | NA | 43 | NA | NA | 68.92 | NA | Oxiracetam | iv, qd, 4g | Nimodipine | po, tid, 40mg | ①③ | 2 |
| Zhou YD2011 | 33 | NA | NA | 32 | NA | NA | 62.1±3.1 | NA | Oxiracetam | po, tid, 0.8g | placebo | NA | ① | 12 |
| Bottini G 1992 | 35 | 11 | 24 | 30 | 17 | 13 | 71 | NA | Oxiracetam | po, bid, 0.8g | placebo | NA | ③ | 12 |
| Dysken MW1989 | 17 | NA | NA | 17 | NA | NA | 74±8 | NA | Oxiracetam | po, bid, 0.4g | placebo | NA | ③ | 12 |
| Maina G 1989 | 145 | NA | NA | 144 | NA | NA | 73 | NA | Oxiracetam | po, bid, 0.8g | placebo | NA | ③ | 12 |
| Villardital 1992 | 30 | 15 | 15 | 30 | 21 | 9 | 71.7±1.3 | 67.8±1.5 | Oxiracetam | po, bid, 0.8g | placebo | NA | ③ | 12 |
| Bo YQ2012 | 56 | 31 | 25 | 56 | 34 | 22 | 65.2±8.5 | 64.3±8.1 | Donepezil | po, qd, 5mg | no-treatment | NA | ①②③ | 12 |
| Chen GQ2018 | 75 | NA | NA | 75 | NA | NA | 72.15±6.33 | NA | Donepezil | po, qd, 5mg | no-treatment | NA | ①② | 8 |
| Chen SQ2007 | 60 | 40 | 20 | 60 | 40 | 20 | 78.8±6.2 | 78.1±7.2 | Donepezil | po, qd, 5mg | Xuesaitong | po, tid, 80mg | ①③ | 12 |
| Shan ZY2011 | 40 | 28 | 12 | 40 | 27 | 13 | 63.4±10.8 | 64.5±12.8 | Donepezil | po, qd, 5mg | no-treatment | NA | ①②③ | 8 |
| Du CB2022 | 62 | 35 | 27 | 62 | 36 | 26 | 65.45±2.69 | 65.42±2.65 | Donepezil | po, qd, 5mg | no-treatment | NA | ①② | 8 |
| Gao CY2012 | 51 | 28 | 23 | 47 | 25 | 22 | 64.3±8.2 | 65.1±8.4 | Donepezil | po, qd, 5mg | no-treatment | NA | ①② | 12 |
| Hao WL2010 | 33 | 18 | 15 | 30 | 17 | 13 | 63.4±10.8 | 64.1±12.8 | Donepezil | po, qd, 5mg | no-treatment | NA | ① | 8 |
| Hu DR2013 | 42 | NA | NA | 42 | NA | NA | NA | NA | Donepezil | po, qd, 5mg | Nimodipine | po, tid, 30mg | ① | 12 |
| Kong YN2007 | 22 | 17 | 5 | 22 | 16 | 6 | 63.6 | 64.4 | Donepezil | po, qd, 5mg | placebo | NA | ② | 24 |
| Li G2019 | 24 | 15 | 9 | 24 | 13 | 11 | 52.4±5.4 | 51.9±5.7 | Donepezil | po, qd, 5mg | no-treatment | NA | ① | 8 |
| Li N2005 | 30 | 18 | 12 | 30 | 20 | 10 | 64.3±6.9 | 65.1±7.9 | Donepezil | po, qd, 5mg | Piracetam | po, tid, 1.2g | ①③ | 8 |
| Li XL2016 | 28 | 16 | 12 | 28 | 15 | 13 | 62.0±1.5 | 61.0±2.0 | Donepezil | po, qd, 5mg | no-treatment | NA | ① | 12 |
| Lin JY2007 | 52 | NA | NA | 52 | NA | NA | NA | NA | Donepezil | po, qd, 5mg | Piracetam | po, tid, 0.4g | ①③ | 12 |
| Pan D2014 | 35 | NA | NA | 35 | NA | NA | NA | NA | Donepezil | po, qd, 5mg | no-treatment | NA | ①②③ | 8 |
| Qiao Y2017 | 41 | 21 | 20 | 41 | 22 | 19 | 59.2±10.5 | 58.6±10.7 | Donepezil | po, qd, 5mg | Xuesaitong | po, tid, 80mg | ①②③ | 12 |
| Qiu YH2011 | 40 | 24 | 16 | 40 | 22 | 18 | 57.89±1.42 | 57.36±1.12 | Donepezil | po, qd, 5mg | no-treatment | NA | ①② | 8 |
| Ren XY2004 | 30 | NA | NA | 30 | NA | NA | NA | NA | Donepezil | po, qd, 5mg | Piracetam | po, tid, 800mg | ①③ | 12 |
| Ren YF2018 | 40 | 22 | 18 | 40 | 19 | 21 | 63.0±5.0 | 62.5±5.0 | Donepezil | po, qd, 5mg | no-treatment | NA | ① | 12 |
| Rong JC2011 | 31 | NA | NA | 31 | NA | NA | NA | NA | Donepezil | po, qd, 5mg | Piracetam | po, tid, 800mg | ① | 16 |
| Tan AX2007 | 30 | 20 | 10 | 28 | 16 | 12 | 67.6±5.3 | 66.6±7.3 | Donepezil | po, qd, 5mg | Piracetam | po, tid, 800mg | ①②③ | 12 |
| Wang XW2010 | 39 | 35 | 4 | 39 | 37 | 2 | 73.03±20.34 | 75.82±17.98 | Donepezil | po, qd, 5mg | Xuesaitong | po, tid, 80mg | ①②③ | 12 |
| Wang Y2006 | 30 | 20 | 10 | 30 | 19 | 11 | 71.2±7.7 | 72.3±6.8 | Donepezil | po, qd, 5mg | Piracetam | po, tid, 800mg | ①②③ | 12 |
| Wen K2006 | 30 | 21 | 9 | 28 | 22 | 6 | 74.67 | 72.63 | Donepezil | po, qd, 5mg | Piracetam | po, tid, 800mg | ①③ | 4 |
| Wu LL2022 | 42 | 22 | 20 | 42 | 23 | 19 | 66.42±2.72 | 66.81±2.67 | Donepezil | po, qd, 5mg | Nicergoline | po, tid, 20mg | ①③ | 4 |
| Yao HM2010 | 41 | 25 | 16 | 41 | 23 | 18 | 67.6±11.5 | 69.4±12.2 | Donepezil | po, qd, 5mg | Nimodipine | po, tid, 30mg | ①② | 12 |
| Zhang SH2007 | 30 | 20 | 10 | 32 | 22 | 10 | 78±6 | 77±7 | Donepezil | po, qd, 5mg | placebo | NA | ①②③ | 12 |
| Zhang WX2014 | 47 | NA | NA | 47 | NA | NA | NA | NA | Donepezil | po, qd, 5mg | Xuesaitong | po, tid, 80mg | ①②③ | 12 |
| Black S 2003 | 206 | 107 | 99 | 199 | 115 | 84 | 73.9±0.6 | 74.2±0.6 | Donepezil | po, qd, 5mg | placebo | NA | ③ | 24 |
| Dichgans M 2008 | 86 | 40 | 46 | 82 | 50 | 32 | 53.8±0.9 | 55.8±0.9 | Donepezil | po, qd, 5mg | placebo | NA | ③ | 18 |
| Gustavo C 2005 | 421 | 241 | 180 | 392 | 220 | 172 | 74.2±0.4 | 74.3±0.4 | Donepezil | po, qd, 5mg | placebo | NA | ③ | 24 |
| Gustavo C 2005arm2 | 406 | 241 | 165 | NA | NA | NA | NA | NA | Donepezil | po, qd, 10mg | NA | NA | ③ | 24 |
| Kavirajan H 2007 | 406 | NA | NA | 568 | NA | NA | NA | NA | Donepezil | po, qd, 5mg | Galantamine | po, bid, 8mg | ③ | 28 |
| Kavirajan H 2007arm2 | 421 | NA | NA | 365 | NA | NA | NA | NA | Donepezil | po, qd, 10mg | Riv,astigmine | po, bid, 6mg | ③ | 28 |
| Kavirajan H 2007arm3 | NA | NA | NA | 460 | NA | NA | NA | NA | Donepezil | po, qd, 5mg | Memantine | po, qd, 20mg | ③ | 28 |
| Pratt RD 2002 | 307 | 184 | 123 | 290 | 162 | 128 | 74.0±0.5 | 74.5±0.5 | Donepezil | po, qd, 5mg | placebo | NA | ③ | 24 |
| Roman G 2010 | 648 | NA | NA | 326 | NA | NA | 73 | | Donepezil | po, qd, 5mg | placebo | NA | ③ | 24 |
| Román GC 2005 | 421 | 241 | 180 | 392 | 220 | 172 | 74.8±0.4 | 74.3±0.4 | Donepezil | po, qd, 10mg | placebo | NA | ③ | 24 |
| Román GC 2010 | 648 | 398 | 250 | 326 | 176 | 150 | 73.4±0.4 | 72.3±0.5 | Donepezil | po, qd, 5mg | placebo | NA | ③ | 24 |
| Wilkinson D 2003 | 215 | 134 | 81 | 193 | 105 | 88 | 75.7±0.6 | 74.4±0.6 | Donepezil | po, qd, 5mg | placebo | NA | ③ | 24 |
| Wilkinson D 2010 | 584 | 237 | 247 | 301 | 128 | 173 | 74.5±0.5 | 74.6±0.5 | Donepezil | po, qd, 5mg | placebo | NA | ③ | 66 |
| Chen RP2014 | 60 | 38 | 22 | 60 | 39 | 21 | 64.5 | 63.8 | Galantamine | po, bid, 5mg | Cerebrolysin | iv, qd, 10ml | ①② | 12 |
| Auchus AP 2007 | 396 | 247 | 149 | 390 | 256 | 134 | 72.3±9.0 | 72.2±8.8 | Galantamine | po, bid, 4mg | placebo | NA | ③ | 26 |
| Erkinjuntti T 2002 | 396 | 207 | 189 | 196 | 105 | 91 | 75.0±6.84 | 75.2±7.32 | Galantamine | po, bid, 4mg | placebo | NA | ③ | 24 |
| Chen H2012 | 30 | 19 | 11 | 30 | 18 | 12 | 64.84±7.12 | 64.23±6.98 | Nicergoline | po, bid, 30mg | Huperzine A | po, bid, 0.2mg | ① | 16 |
| Lou FJ2010 | 42 | 22 | 20 | 42 | 25 | 17 | 54.5±6 | 55.8±9 | Nicergoline | po, tid, 10mg | Oxiracetam | iv, qd, 4.0g | ③ | 12 |
| Lou FJ2010arm2 | NA | NA | NA | 42 | 24 | 18 |  | 56.5±6 | Nicergoline | po, tid, 10mg | placebo | NA |  | 12 |
| Lu JH2001 | 50 | NA | NA | 53 | NA | NA | NA | NA | Nicergoline | po, bid, 30mg | Aniracetam | po, bid, 200mg | ①②③ | 12 |
| Song WJ2006 | 55 | 35 | 20 | 57 | 39 | 18 | NA | NA | Nicergoline | po, bid, 30mg | Aniracetam | po, bid, 200mg | ①② | 12 |
| Tian FC2007 | 32 | 18 | 14 | 32 | 17 | 15 | 66±8 | 64±9 | Nicergoline | po, tid, 20mg | Piracetam | po, bid, 900mg | ①③ | 24 |
| Xu HH2009 | 49 | 26 | 23 | 49 | 28 | 21 | NA | NA | Nicergoline | po, tid, 10mg | Piracetam | po, tid, 1.2g | ①②③ | 12 |
| Xu SY2013 | 38 | 20 | 18 | 37 | 21 | 17 | NA | NA | Nicergoline | po, tid, 20mg | Piracetam | po, tid, 1.5g | ①② | 12 |
| Zhang YH2008 | 29 | 14 | 15 | 28 | 15 | 13 | NA | NA | Nicergoline | po, qd, 30mg | Aniracetam | po, bid, 200mg | ①② | 12 |
| Bin M2008 | 34 | NA | NA | 32 | NA | NA | NA | NA | Nimodipine | po, bid, 30mg | no-treatment | NA | ①② | 4 |
| Chen GM2012 | 40 | 22 | 18 | 40 | 21 | 19 | 64.2±2.11 | 52.2±2.91 | Nimodipine | po, tid, 30mg | no-treatment | NA | ① | 24 |
| Chen XY2009 | 27 | NA | NA | 26 | NA | NA | NA | NA | Nimodipine | po, tid, 40mg | no-treatment | NA | ① | 12 |
| Cheng L2013 | 29 | 18 | 11 | 29 | 16 | 13 | 57.8 | 59.4 | Nimodipine | po, tid, 30mg | no-treatment | NA | ①② | 12 |
| Du XH2016 | 37 | 18 | 19 | 37 | 20 | 17 | 67.2±4.3 | 64.9±3.7 | Nimodipine | po, tid, 30mg | no-treatment | NA | ① | 12 |
| Guo LL2006 | 54 | NA | NA | 52 | NA | NA | NA | NA | Nimodipine | po, tid, 40mg | no-treatment | NA | ① | 12 |
| Hou LF2015 | 40 | NA | NA | 40 | NA | NA | NA | NA | Nimodipine | po, tid, 40mg | Oxiracetam | iv, qd, 4.0g | ① | 3 |
| Huang YX2010 | 30 | NA | NA | 30 | NA | NA | NA | NA | Nimodipine | po, tid, 30mg | Piracetam | po, tid, 0.8mg | ① | 12 |
| Jin LY2013 | 39 | NA | NA | 39 | NA | NA | NA | NA | Nimodipine | po, tid, 30mg | no-treatment | NA | ①②③ | 12 |
| Li HM2019 | 100 | 65 | 35 | 100 | 67 | 33 | 66.50±6.13 | 65.42±5.93 | Nimodipine | po, tid, 40mg | Butylphthalide | po, tid, 100 mg | ①③ | 24 |
| Niu XJ2016 | 40 | 24 | 16 | 40 | 23 | 17 | 59.85±2.21 | 59.64±2.13 | Nimodipine | po, tid, 40mg | no-treatment | NA | ① | 24 |
| Sui CF2021 | 59 | 31 | 28 | 59 | 30 | 29 | 76.5±5.9 | 77.6±6.0 | Nimodipine | po, tid, 40mg | Co-dergocrine Mesyiate | po, tid, 2mg | ①②③ | 12 |
| Wang SF2012 | 48 | 29 | 19 | 44 | 26 | 18 | 64.2±5.1 | 64.7±4.8 | Nimodipine | po, tid, 30mg | no-treatment | NA | ①③ | 8 |
| Wang YF2009 | 60 | 38 | 22 | 60 | 39 | 21 | 65.8±7.6 | 65.4±7.9 | Nimodipine | po, tid, 30mg | Donepezil | po, qd, 5mg | ①③ | 24 |
| Wang ZG2009 | 20 | NA | NA | 16 | NA | NA | NA | NA | Nimodipine | po, tid, 30mg | Donepezil | po, qd, 5mg | ①②③ | 12 |
| Wang ZF2016 | 54 | 29 | 25 | 54 | 31 | 23 | 63.7±7.6 | 64.2±8.2 | Nimodipine | po, bid, 60mg | Donepezil | po, qd, 5mg | ① | 12 |
| Xu XY2017 | 40 | 23 | 17 | 40 | 22 | 18 | 53.6±4.7 | 53.9±4.8 | Nimodipine | po, tid, 50mg | Butylphthalide | po, tid, 400mg | ①③ | 24 |
| Zhang XZ2018 | 50 | 28 | 22 | 50 | 27 | 23 | 72.3±5.5 | 72.0±5.0 | Nimodipine | po, tid, 40mg | no-treatment | NA | ①② | 12 |
| Zhou Y2021 | 110 | 61 | 49 | 115 | 64 | 52 | 69.12±7.36 | 69.51±7.59 | Nimodipine | po, qd, 5mg | Donepezil | po, tid, 5mg | ①② | 12 |
| Pantoni L 2000 | 83 | 43 | 40 | 84 | 42 | 42 | 73.0±8.13 | 74.5±6.40 | Nimodipine | po, tid, 30mg | placebo | NA | ③ | 24 |
| Pantoni L 2005 | 121 | 70 | 41 | 109 | 67 | 42 | 75.2±6.1 | 75.4±6.0 | Nimodipine | po, tid, 30mg | placebo | NA | ①③ | 52 |
| Chen YH2020 | 25 | 16 | 9 | 25 | 17 | 8 | 71.32±3.47 | 70.15±3.26 | Memantine | po, qd, 5mg | Piracetam | po, qd, 0.8g | ① | 24 |
| Du LZ2020 | 20 | 11 | 9 | 20 | 12 | 8 | 70.2±6.1 | 70.0±6.2 | Memantine | po, qd, 5mg | Donepezil | po, qd, 5mg | ①②③ | 12 |
| He D2018 | 34 | 25 | 9 | 36 | 26 | 10 | 72.97±4.11 | 74.95±4.82 | Memantine | po, qd, 5mg | Piracetam | po, qd, 0.8g | ③ | 24 |
| Li YY2009 | 31 | 27 | 4 | 30 | 27 | 3 | 72±lO | 7l±9 | Memantine | po, qd, 5mg | Piracetam | po, tid, 0.8g | ① | 12 |
| Ouyang XC2012 | 30 | 24 | 6 | 30 | 25 | 5 | 72.8±4.8 | 74.5±5.6 | Memantine | po, qd, 5mg | Piracetam | po, tid, 0.8g | ①② | 24 |
| Yao MR2015 | 35 | 26 | 9 | 35 | 27 | 8 | 73.9±4.7 | 74.6±5.7 | Memantine | po, qd, 5mg | placebo | po, qd, 5mg | ①② | 24 |
| Orgogozo JM 2002 | 93 | NA | NA | 95 | NA | NA | 76.6±6.5 | 76.1±6.86 | Memantine | po, qd, 5mg | placebo | po, qd, 5mg | ③ | 28 |
| Wilcock G 2002 | 277 | 143 | 134 | 271 | 138 | 133 | 77.2±6.9 | 77.6±7.0 | Memantine | po, qd, 5mg | placebo | po, qd, 5mg | ③ | 28 |
| Xiao J 2004 | 23 | 14 | 9 | 23 | 14 | 9 | 74.3 | 79.1 | Riv,astigmine | po, qd, 3mg | Piracetam | po, tid, 0.8g | ①③ | 12 |
| Zhong XD 2021 | 30 | 16 | 14 | 30 | 19 | 11 | 63.26±7.91 | 63.33±9.82 | Riv,astigmine | po, qd, 3mg | Piracetam | po, tid, 0.8g | ①② | 12 |
| Zhong XD 2021arm2 | NA | NA | NA | 30 | 18 | 12 | NA | 63.78±8.32 | NA | NA | Co-dergocrine Mesyiate | po, tid,1mg | ①② | 12 |
| Zhong XD 2021arm3 | NA | NA | NA | 30 | 17 | 13 | NA | 63.90±6.32 | NA | NA | Donepezil | po, tid, 5mg | ①② | 12 |
| Zhong XD 2021arm4 | NA | NA | NA | 30 | 20 | 10 | NA | 63.70±5.44 | NA | NA | Memantine | po, qd, 5mg | ①② | 12 |
| Ballard C 2008 | 365 | 223 | 142 | 345 | 219 | 126 | 72.9±8.3 | 72.7±7.6 | Riv,astigmine | po, bid, 1.5mg | placebo | NA | ③ | 24 |
| Mok V 2007 | 20 | 7 | 13 | 20 | 9 | 11 | 75.7±5.1 | 74.1±6.6 | Riv,astigmine | po, bid, 1.5mg | placebo | NA | ③ | 26 |
| Moretti R 2002 | 8 | 5 | 3 | 8 | 5 | 3 | 72.23±3.46 | 72.45±1.21 | Riv,astigmine | po, qd, 3mg | placebo | NA | ③ | 88 |
| Moretti R 2005 | 104 | NA | NA | 104 | NA | NA | NA | NA | Riv,astigmine | po, qd, 3mg | placebo | NA | ③ | 48 |
| Moretti R2004 | 8 | NA | NA | 8 | NA | NA | NA | NA | Riv,astigmine | po, qd, 3mg | placebo | NA | ③ | 88 |
| Bai XY2019 | 39 | 21 | 18 | 39 | 20 | 19 | 61.21±9.87 | 61.98±8.94 | Butylphthalide | po, tid, 200mg | Nimodipine | po, tid, 30mg | ③ | 4 |
| Chang LX2019 | 56 | 36 | 20 | 55 | 34 | 21 | 68.66±6.21 | 68.33±6.12 | Butylphthalide | po, tid, 200mg | Piracetam | po, tid, 0.8g | ① | 8 |
| Chen GF2012 | 34 | 23 | 11 | 33 | 22 | 11 | 63.82±10.26 | 64.67±10.58 | Butylphthalide | po, tid, 200mg | no-treatment | NA | ①③ | 12 |
| Cheng WY2016 | 48 | 31 | 17 | 48 | 32 | 16 | 65.2±8.9 | 64.6±9.2 | Butylphthalide | po, tid, 200mg | Nimodipine | po, tid, 40mg | ①②③ | 24 |
| He XZ2016 | 34 | 25 | 9 | 34 | 28 | 6 | 68.7±14.3 | 66.2±13.6 | Butylphthalide | po, tid, 200mg | no-treatment | NA | ① | 12 |
| Hou DR2009 | 30 | 18 | 12 | 30 | 17 | 13 | 66.1±8.2 | 65.9±7.7 | Butylphthalide | po, tid, 200mg | Piracetam | po, tid, 0.8g | ①② | 8 |
| Hu H2017 | 41 | 25 | 16 | 41 | 26 | 15 | 66.5±5.3 | 66.9±5.4 | Butylphthalide | po, tid, 200mg | no-treatment | NA | ① | 8 |
| Li HM2019 | 100 | 65 | 35 | 100 | 67 | 33 | 66.50±6.13 | 65.42±5.93 | Butylphthalide | po, tid, 200mg | Nimodipine | po, tid, 40mg | ①③ | 24 |
| Li S2015 | 49 | 27 | 22 | 49 | 25 | 24 | NA | NA | Butylphthalide | po, tid, 200mg | no-treatment | NA | ①③ | 3 |
| Liu C2013 | 50 | 34 | 16 | 50 | 36 | 14 | 76.69±18.5 | 77.83±17.25 | Butylphthalide | po, tid, 200mg | Co-dergocrine Mesyiate | po, tid, 1mg | ①③ | 12 |
| Liu FQ2015 | 49 | 27 | 22 | 49 | 29 | 20 | 63.7±8.3 | 63.5±7.9 | Butylphthalide | po, tid, 200mg | Piracetam | po, tid, 0.8g | ①② | 8 |
| Liu J2020 | 66 | 38 | 28 | 66 | 39 | 27 | 73.87±6.47 | 73.56±6.78 | Butylphthalide | po, tid, 200mg | Piracetam | po, tid, 1.2g | ① | 12 |
| Liu XL2015 | 42 | 28 | 14 | 42 | 30 | 12 | 68.9±4.5 | 69.4±5.4 | Butylphthalide | po, tid, 200mg | no-treatment | NA | ①③ | 12 |
| Long CY2012 | 42 | 28 | 14 | 40 | 26 | 14 | 63.5±4.3 | 62.1±3.9 | Butylphthalide | po, tid, 200mg | no-treatment | NA | ①③ | 12 |
| Lu HL2011 | 50 | 28 | 22 | 50 | 27 | 23 | 65.2 | 64.6 | Butylphthalide | po, tid, 200mg | Nimodipine | po, tid, 30mg | ①②③ | 12 |
| Lv CL2015 | 48 | NA | NA | 48 | NA | NA | NA | NA | Butylphthalide | po, tid, 200mg | no-treatment | NA | ①② | 4 |
| Ma J2017 | 61 | 37 | 24 | 61 | 35 | 26 | 68.2±2.7 | 68.5±2.6 | Butylphthalide | po, tid, 200mg | placebo | NA | ① | 12 |
| Shao XP2013 | 40 | 24 | 16 | 40 | 27 | 13 | 68.76±5.89 | 67.01±4.67 | Butylphthalide | po, tid, 200mg | Nimodipine | po, tid, 30mg | ①③ | 12 |
| Shao XP2013arm2 | NA | NA | NA | 40 | 23 | 17 | 68.76±5.89 | 69.49±5.91 | Butylphthalide | po, tid, 200mg | Piracetam | po, tid, 0.8g | ①③ | 12 |
| Sheng GL2015 | 49 | 26 | 23 | 49 | 28 | 21 | 56.5±5.3 | 58.7±5.1 | Butylphthalide | po, tid, 400mg | Nimodipine | po, tid, 50mg | ①③ | 24 |
| Su XD2015 | 31 | 19 | 12 | 31 | 21 | 10 | 63.0±5.2 | 62.0±7.9 | Butylphthalide | po, tid, 600mg | Nimodipine | po, bid, 120mg | ①③ | 24 |
| Sun LY2013 | 54 | 30 | 24 | 58 | 31 | 27 | 70.8±5.2 | 71.6±5.5 | Butylphthalide | po, tid, 200mg | no-treatment | NA | ①③ | 12 |
| Wang QY2014 | 42 | 26 | 16 | 42 | 25 | 17 | 67.6±5.5 | 67.8±5.6 | Butylphthalide | po, tid, 200mg | Co-dergocrine Mesyiate | po, tid, 1mg | ① | 12 |
| Wang S2021 | 40 | 22 | 18 | 40 | 24 | 16 | 61.20±7.60 | 61.30±8.30 | Butylphthalide | po, tid, 200mg | Nimodipine | po, tid, 30mg | ③ | 4 |
| Wang YJ2013 | 50 | NA | NA | 50 | NA | NA | NA | NA | Butylphthalide | iv, bid, 25mg | Oxiracetam | iv, qd, 4.0g | ①②③ | 4 |
| Wu J2012 | 31 | 14 | 17 | 31 | 16 | 15 | 65.9±6.8 | 66.5±6.7 | Butylphthalide | po, tid, 200mg | placebo | NA | ①②③ | 12 |
| Xia DY2013 | 30 | 16 | 14 | 30 | 17 | 13 | NA | NA | Butylphthalide | po, tid, 200mg | Nimodipine | po, tid, 40mg | ①③ | 12 |
| Xiao YZ2019 | 35 | 25 | 10 | 35 | 22 | 13 | 59.24±1.12 | 59.10±1.10 | Butylphthalide | iv, bid, 25mg | Oxiracetam | iv, qd, 4.0g | ①③ | 2 |
| Xu F2015 | 15 | 10 | 5 | 15 | 9 | 6 | 66.6±5.5 | 65.8±5.2 | Butylphthalide | po, tid, 200mg | no-treatment | NA | ①③ | 12 |
| Yang Y2020 | 67 | 27 | 42 | 69 | 37 | 32 | 62.25±5.31 | 63.25±5.51 | Butylphthalide | po, tid, 200mg | Nimodipine | po, tid, 40mg | ① | 24 |
| Yin YB2018 | 43 | 27 | 16 | 43 | 28 | 15 | 70.1±10.7 | 69.2±10.8 | Butylphthalide | po, tid, 200mg | Piracetam | po, tid, 0.8g | ① | 8 |
| Zhang L2018 | 30 | 18 | 12 | 30 | 17 | 13 | 62.1±5.5 | 62.0±5.7 | Butylphthalide | po, tid, 200mg | no-treatment | NA | ① | 12 |
| Wang J2016 | 36 | 21 | 15 | 36 | 20 | 16 | 64.5±2.5 | 64.7±2.3 | Cerebrolysin | iv, qd, 10ml | Citicoline | iv, qd, 0.25g | ③ | 3 |
| Xue H2016 | 40 | 21 | 19 | 40 | 22 | 18 | 65.83±3.79 | 65.27±3.18 | Cerebrolysin | iv, qd, 10ml | Citicoline | iv, qd, 0.25g | ③ | 3 |
| Guekht AB 2011 | 117 | 39 | 78 | 115 | 48 | 67 | 67.1±8.0 | 67.6±8.0 | Cerebrolysin | iv, qd, 20ml | placebo | NA | ③ | 24 |
| Muresanu DF 2008 | 10 | 5 | 5 | 31 | 16 | 15 | 70.3±1.9 | 72.1±3.1 | Cerebrolysin | iv, qd, 10ml | placebo | NA | ③ | 4 |
| Muresanu DF 2010 | 9 | 5 | 4 | 24 | 11 | 1 | 71.89± 3.52 | 71.50±2.21 | Cerebrolysin | iv, qd, 10ml | placebo | NA | ③ | 12 |
| Hu F2017 | 45 | 23 | 22 | 45 | 21 | 24 | 68.3±3.1 | 68.0±1.3 | Huperzine A | po, bid, 0.1 mg | no-treatment | NA | ①② | 24 |
| Liu H2015 | 44 | 27 | 17 | 44 | 29 | 15 | 68.3±3.2 | 70.3±2.8 | Huperzine A | po, bid, 0.1 mg | Donepezil | po, qd, 5mg | ③ | 6 |
| Liu L2015 | 22 | NA | NA | 22 | NA | NA | NA | NA | Huperzine A | po, bid, 0.2mg | Oxiracetam | po, tid, 0.8g | ①② | 50 |
| Liu L2015arm2 | NA | NA | NA | 22 | NA | NA | NA | NA | NA | NA | Nimodipine | po, tid, 70mg | ①② | 50 |
| Wang FL2011 | 30 | 20 | 10 | 30 | 21 | 9 | 70.9±5.2 | 71.6±6.3 | Huperzine A | po, tid, 0.1 mg | placebo | NA | ①② | 12 |
| Wang RP2004 | 36 | 24 | 12 | 32 | 21 | 11 | 64.0±5.9 | 64.8±6.1 | Huperzine A | po, bid, 0.1 mg | placebo | NA | ①②③ | 24 |
| Wang WL2010 | 21 | 17 | 4 | 21 | 13 | 8 | 81±3.5 | 84±5.4 | Huperzine A | po, bid, 0.1 mg | Donepezil | po, qd, 5mg | ①②③ | 48 |
| Wang YQ2000 | 17 | NA | NA | 18 | NA | NA | 75.3±6.2 | 75.8±7.3 | Huperzine A | po, bid, 0.1 mg | Donepezil | po, tid, 0.8g | ③ | 8 |
| Weng QL2011 | 37 | 19 | 18 | 45 | 25 | 20 | 73.1±10.0 | 72.1±9.5 | Huperzine A | po, tid, 0.0.5mg | Piracetam | po, tid, 0.4g | ①③ | 52 |
| Weng QL2011arm2 | 36 | 19 | 17 | NA | NA | NA | 73.3±8.8 | NA | Huperzine A | po, tid, 0.1 mg | NA | NA | ①③ |  |
| Weng QL2011arm3 | 38 | 21 | 17 | NA | NA | NA | 72.0±8.1 | NA | Huperzine A | po, tid, 0.15 mg | NA | NA | ①③ |  |
| Xu ZJ2009 | 25 | 17 | 8 | 26 | 16 | 10 | 72.08±11.86 | 67.864±13.22 | Huperzine A | po, bid, 0.1 mg | placebo | NA | ②③ | 24 |
| Xu ZQ2009 | 39 | 24 | 15 | 39 | 26 | 13 | 71.8±7.2 | 72.3±6.9 | Huperzine A | po, bid, 0.1 mg | placebo | NA | ①②③ | 12 |
| Zhang HP2013 | 40 | 27 | 13 | 40 | 21 | 19 | 67±4.5 | 65±5.6 | Huperzine A | po, bid, 0.1 mg | Piracetam | po, tid, 0.8g | ①② | 8 |
| Zhang SG2011 | 40 | 20 | 20 | 40 | 22 | 18 | 58.37±6.91 | 57.52±9.30 | Huperzine A | po, bid, 0.1 mg | Nimodipine | po, tid, 30mg | ①② | 12 |
| Zhao M2007 | 31 | 16 | 15 | 33 | 19 | 14 | 67.89±6.58 | 68.12±6.2 | Huperzine A | po, bid, 0.1 mg | Donepezil | po, qd, 5mg | ①②③ | 6 |
| Zheng GL2006 | 40 | 24 | 16 | 40 | 22 | 18 | 65.8±7.6 | 67.4±6.9 | Huperzine A | po, bid, 0.1 mg | Piracetam | po, tid, 0.8g | ①③ | 24 |
| Zhong ZG2004 | 29 | 17 | 12 | 28 | 18 | 10 | 72.4±5.8 | 74.1±6.2 | Huperzine A | po, bid, 0.1 mg | no-treatment | NA | ①②③ | 12 |
| Xu ZQ 2012 | 39 | 24 | 15 | 39 | 26 | 13 | 71.8±7.2 | 72.3±6.9 | Huperzine A | po, bid, 0.1 mg | no-treatment | NA | ①②③ | 12 |
| Chen CL2009 | 60 | 31 | 29 | 60 | 33 | 27 | 64±7 | 64±7 | Tongxinluo capsule | po, tid, 1.04g | Donepezil | po, qd, 5mg | ①③ | 12 |
| Hao WP2006 | 50 | 30 | 20 | 50 | 28 | 22 | 70.20±5.24 | 71.30±5.80 | Tongxinluo capsule | po, tid, 2.34g | Piracetam | po, tid, 0.8g | ①③ | 24 |
| Wang LL2005 | 40 | 26 | 14 | 40 | 29 | 11 | 62.78±6.54 | 63.56±6.58 | Tongxinluo capsule | po, tid, 0.78g | placebo | NA | ①③ | 24 |
| Yuan LF2011 | 27 | 14 | 13 | 26 | 14 | 12 | 62±5 | 63±4 | Tongxinluo capsule | po, tid, 1.04g | placebo | NA | ① | 12 |
| Du W2012 | 33 | 22 | 11 | 33 | 21 | 12 | 62.01 | 61.54 | Edaravone | iv, bid, 30ml | no-treatment | NA | ① | 2 |
| Li HY2010 | 30 | NA | NA | 30 | NA | NA | NA | NA | Edaravone | iv, bid, 30ml | no-treatment | NA | ①②③ | 3 |
| Zhang F2010 | 54 | 30 | 24 | 52 | 29 | 23 | 65.01±6.67 | 64.09±7.10 | Edaravone | iv, bid, 30ml | no-treatment | NA | ①③ | 2 |
| Zhang HQ2012 | 35 | 17 | 18 | 35 | 18 | 17 | 63.2±7.4 | 64.7±6.4 | Edaravone | iv, bid, 30ml | Piracetam | po, tid, 0.8g | ① | 2 |
| Zhou GA2012 | 40 | 19 | 22 | 40 | 20 | 20 | 62.1±8.2 | 65.9±5.7 | Edaravone | iv, bid, 30ml | Piracetam | po, tid, 0.8g | ① | 2 |
| Shi J 2020 | 232 | 154 | 78 | 233 | 149 | 84 | 45-85 | 45-85 | Tianzhi granule | po, tid, 5g | Donepezil | po, qd, 5mg | ①③ | 2 |
| Shi J 2020arm2 | NA | NA | NA | 55 | 35 | 20 | NA | 45-82 | Tianzhi granule | po, tid, 5g | placebo | NA | ①③ | 2 |
| Du GY2003 | 100 | NA | NA | 100 | NA | NA | NA | NA | Tianzhi granule | po, tid, 5g | Almitrine Bismesylate Raubasine | po, qd, 30mg | ①③ | 8 |
| Li SL2011 | 61 | NA | NA | 49 | NA | NA | NA | NA | Tianzhi granule | po, tid, 5g | Piracetam | po, tid, 1.8g | ①③ | 4 |
| Li SZ 2011 | 78 | 40 | 38 | 60 | 34 | 26 | 58-89 | 61-90 | Tianzhi granule | po, tid, 5g | placebo | NA | ① | 8 |
| Lian CL 2010 | 50 | 36 | 14 | 50 | 32 | 18 | 53-82 | 51-80 | Tianzhi granule | po, tid, 5g | Piracetam | po, tid, 0.8g | ③ | 8 |
| Liu XP 2009 | 60 | 35 | 25 | 50 | 30 | 20 | 74±6 | 73±6 | Tianzhi granule | po, tid, 5g | Almitrine Bismesylate Raubasine | po, qd, 10mg | ① | 8 |
| Niu QY 2008 | 42 | 27 | 15 | 25 | 15 | 10 | 65.52±8.12 | 66.23±7.76 | Tianzhi granule | po, tid, 5g | Aniracetam | po, tid, 0.5g | ① | 8 |
| Wang SH 2012 | 30 | 19 | 11 | 30 | 18 | 12 | 46-78 | 47-75 | Tianzhi granule | po, tid, 5g | placebo | NA | ①② | 4 |
| Wen GL 2016 | 60 | 28 | 22 | 60 | 24 | 36 | 61.7±3.2 | 60.3±4.5 | Tianzhi granule | po, tid, 5g | Piracetam | po, tid, 0.8g | ① | 12 |
| Wu B 2013 | 30 | 16 | 14 | 30 | 18 | 12 | 73.2±8.5 | 72.6±7.8 | Tianzhi granule | po, tid, 5g | Aniracetam | po, tid, 0.2g | ①② | 8 |
| Wu JT 2011 | 27 | 15 | 12 | 25 | 14 | 11 | 70±5 | 71±4 | Tianzhi granule | po, tid, 5g | Oxiracetam | po, tid, 0.8g | ①② | 12 |
| Yang H 2011 | 32 | NA | NA | 30 | NA | NA | NA | NA | Tianzhi granule | po, tid, 5g | no-treatment | NA | ①③ | 8 |
| Yao JM 2006 | 42 | 22 | 20 | 21 | 8 | 13 | 68.90±7.65 | 68.52±8.08 | Tianzhi granule | po, tid, 5g | Almitrine Bismesylate Raubasine | po, qd, 10mg | ①③ | 8 |
| Zhu AH 2005 | 70 | 44 | 26 | 50 | 31 | 19 | 65.93±8.64 | 68.68±7.53 | Tianzhi granule | po, tid, 5g | Almitrine Bismesylate Raubasine | po, qd, 10mg | ① | 8 |
| *IG: intervention group; CG: control group; ①:MMSE; ②:ADL; ③:adverse effects rate; po,: oral administration; iv,: intravenous injection; qd,: once a day; bid,: twice a day; tid,: three times a day; NA Not applicable | | | | | | | | | | | | | |  |
