# Supplementary material for: Pharmacological treatments for vascular dementia: a systematic review and Bayesian network meta-analysis
Source: Front Pharmacol. 2024 Aug 22;15:1451032. doi: 10.3389/fphar.2024.1451032 (PMC11374729; doi:10.3389/fphar.2024.1451032)
Supplement: Supplementary file 4 [file DataSheet1.docx]

Supplementary Text 1. Search strategies.

1. **PubMed**

(("Dementia, Vascular"[Mesh]) OR ("Vascular Dementias"[Title/Abstract]) OR ("Vascular Dementia"[Title/Abstract])) AND (("Drugs, Chinese Herbal"[Mesh]) OR ("Herbal Medicine"[Mesh]) OR ("Herbal Medicine"[Title/Abstract]) OR ("Medicine, Chinese Traditional"[Mesh]) OR ("Chinese Traditional Medicine"[Title/Abstract]) OR ("Pharmaceutical Preparations"[Mesh]) OR ("Drug Therapy"[Mesh]) OR ("Injections, Intravenous"[Mesh]) OR ("Idebenone"[Title/Abstract]) OR ("Oxiracetam"[Title/Abstract]) OR ("Acetaminophen"[MeSH Terms] OR "Acetaminophen"[Title/Abstract]) OR ("Donepezil"[MeSH Terms] OR "Donepezil"[Title/Abstract]) OR ("Metformin"[MeSH Terms] OR "Metformin"[Title/Abstract]) OR ("Galantamine"[MeSH Terms] OR "Galantamine"[Title/Abstract]) OR ("Rivastigmine"[MeSH Terms] OR "Rivastigmine"[Title/Abstract]) OR ("Cerebrolysin"[Title/Abstract]) OR ("Nicergoline"[MeSH Terms] OR "Nicergoline"[Title/Abstract]) OR ("Nimodipine"[MeSH Terms] OR "Nimodipine"[Title/Abstract]) OR ("Pimavanserin"[Title/Abstract]) OR ("Huperzine A"[Title/Abstract]) OR ("Dronabinol"[MeSH Terms] OR "Tetrahydrocannabinol"[Title/Abstract]) OR ("Hydroxymethylglutaryl-CoA Reductase Inhibitors"[MeSH Terms] OR "Statins"[Title/Abstract]) OR ("Memantine"[MeSH Terms] OR "Memantine"[Title/Abstract]) OR ("Acetylcysteine"[MeSH Terms] OR "N-Acetylcysteine"[Title/Abstract]) OR ("Pentoxifylline"[MeSH Terms] OR "Pentoxifylline"[Title/Abstract]))

1. **Embase**

('vascular dementia'/exp OR 'vascular dementia' OR 'vascular dementias') AND ('chinese traditional medicine'/exp OR 'chinese traditional medicine' OR 'herbal drug'/exp OR 'herbal drug' OR 'herbal medicine' OR 'drug therapy'/exp OR 'drug therapy' OR 'intravenous drug administration'/exp OR 'intravenous drug administration' OR 'idebenone'/exp OR 'idebenone' OR 'oxiracetam'/exp OR 'oxiracetam' OR 'acetaminophen'/exp OR 'acetaminophen' OR 'donepezil'/exp OR 'donepezil' OR 'metformin'/exp OR 'metformin' OR 'galantamine'/exp OR 'galantamine' OR 'rivastigmine'/exp OR 'rivastigmine' OR 'cerebrolysin'/exp OR 'cerebrolysin' OR 'nicergoline'/exp OR 'nicergoline' OR 'nimodipine'/exp OR 'nimodipine' OR 'pimavanserin'/exp OR 'pimavanserin' OR 'huperzine a'/exp OR 'huperzine a' OR 'dronabinol'/exp OR 'dronabinol' OR 'hydroxymethylglutaryl-coa reductase inhibitor' OR 'statin' OR 'memantine'/exp OR 'memantine' OR 'acetylcysteine'/exp OR 'n-acetylcysteine' OR 'pentoxifylline'/exp OR 'pentoxifylline')

1. **Cochrane Library**

("Vascular Dementia" OR "Vascular Dementias" OR"VD"OR"VaD") AND

("Chinese Traditional Medicine" OR "Chinese Herbal Drugs" OR "Herbal Medicine" OR "Pharmaceutical Preparations" OR "Drug Therapy" OR "Intravenous Drug Administration") NOT"Alzheimer's Disease"

1. **Web of Science**

TS=("Dementia, Vascular" OR "Vascular Dementias" OR "Vascular Dementia")

AND

TS=("Drugs, Chinese Herbal" OR "Herbal Medicine" OR "Medicine, Chinese Traditional" OR "Chinese Traditional Medicine" OR "Pharmaceutical Preparations" OR "Drug Therapy" OR "Injections, Intravenous" OR "Idebenone" OR "Oxiracetam" OR "Acetaminophen" OR "Donepezil" OR "Metformin" OR "Galantamine" OR "Rivastigmine" OR "Cerebrolysin" OR "Nicergoline" OR "Nimodipine" OR "Pimavanserin" OR "Huperzine A" OR "Dronabinol" OR "Tetrahydrocannabinol" OR "Statins" OR "Memantine" OR "N-Acetylcysteine" OR "Pentoxifylline")

1. **OPENGREY**

(Idebenone or Oxiracetam or Donepezil or Galantamine or Rivastigmine or Cerebrolysin or Nicergoline or Nimodipine or statins or Huperzine A or Atorvastatin or Rosuvastatin or Simvastatin or Memantine or Breviscapine or Butylphthalide or Naoxintong or Tianzhi granule or Tongxinluo capsule or Edaravone or Yinxing or Vinpocetine or Piracetam or Aniracetam or Co-dergocrine Mesyiate or Citicoline or Xuesaitong or Almitrine Bismesylate Raubasine) And (Vascular Dementia or VD or VaD)

1. **ClinicalTrials.gov:**

Intervention/treatment: Idebenone OR Oxiracetam OR Donepezil OR Galantamine OR Rivastigmine OR Cerebrolysin OR Nicergoline OR Nimodipine OR statins OR statins OR Huperzine A OR Atorvastatin OR Rosuvastatin OR Simvastatin OR Memantine OR Breviscapine OR Butylphthalide OR Naoxintong OR Tianzhi granule OR Tongxinluo capsule OR Edaravone OR Yinxing OR Vinpocetine OR Piracetam OR Aniracetam OR Co-dergocrine Mesyiate OR Citicoline OR Xuesaitong OR Almitrine Bismesylate Raubasine

Outcome Measure: Mini-Mental State Examination OR MMSE OR ADL OR activities of daily living score OR the incidence of adverse reactions OR rate of adverse reactions

Study Type: Interventional (Clinical Trial)

Study Results: With results.

**7.万方**

“血管性痴呆”“血管性认知障碍”和“药物治疗”，“多奈哌齐”“艾地苯醌”,“奥拉西坦”“石杉碱甲”“美金刚”“尼莫地平”, “对乙酰氨基酚”, “加兰他敏”,“卡巴拉汀”, “脑活素”, “尼麦角林”, “尼莫地平”, “他克林”, “他汀”, “阿托伐他汀”, “辛伐他汀”, “瑞舒伐他汀”, “盐酸美金刚”, “乙酰半胱氨酸”, “已酮可可碱”, “脑复康”,“吡拉西坦”,“茴拉西坦”,“阿尼西坦”,“灯盏花素”,“丁苯酞”,“脑心通”,“天智颗粒”,“通心络胶囊”,“依达拉奉”,“银杏”,“长春西汀”,“胞磷胆碱”,“血塞通”,“甲磺酸阿米三嗪萝巴新片”,“都可喜”

("血管性痴呆" OR "血管性认知障碍")

AND

("药物治疗" OR "多奈哌齐" OR "艾地苯醌" OR "奥拉西坦" OR "石杉碱甲" OR "美金刚" OR "尼莫地平" OR "对乙酰氨基酚" OR "加兰他敏" OR "卡巴拉汀" OR "脑活素" OR "尼麦角林" OR "尼莫地平" OR "他克林" OR "他汀" OR "阿托伐他汀" OR "辛伐他汀" OR "瑞舒伐他汀" OR "盐酸美金刚" OR "乙酰半胱氨酸" OR "已酮可可碱" OR "脑复康" OR "吡拉西坦" OR "茴拉西坦" OR "阿尼西坦" OR "灯盏花素" OR "丁苯酞" OR "脑心通" OR "天智颗粒" OR "通心络胶囊" OR "依达拉奉" OR "银杏" OR "长春西汀" OR "胞磷胆碱" OR "血塞通" OR "甲磺酸阿米三嗪萝巴新片" OR "都可喜")

1. **知网**

('血管性痴呆' + '血管性认知障碍') * ('药物治疗' * '多奈哌齐' * '艾地苯醌' * '奥拉西坦' * '石杉碱甲' * '美金刚' * '尼莫地平' * '对乙酰氨基酚' * '加兰他敏' * '卡巴拉汀' * '脑活素' * '尼麦角林' * '尼莫地平' * '他克林' * '他汀' * '阿托伐他汀' * '辛伐他汀' * '瑞舒伐他汀' * '盐酸美金刚' * '乙酰半胱氨酸' * '已酮可可碱' * '脑复康' * '吡拉西坦' * '茴拉西坦' * '阿尼西坦' * '灯盏花素' * '丁苯酞' * '脑心通' * '天智颗粒' * '通心络胶囊' * '依达拉奉' * '银杏' * '长春西汀' * '胞磷胆碱' * '血塞通' * '甲磺酸阿米三嗪萝巴新片' * '都可喜')
